# Dielectric metasurface-assisted terahertz sensing: mechanism, fabrication, and multiscenario applications

**DOI:** 10.1515/nanoph-2024-0573

**Published:** 2025-01-31

**Authors:** Xueer Chen, Shanshan Xin, Qing Liu, Yihan Meng, Daquan Yu, Ming Lun Tseng, Longfang Ye

**Affiliations:** School of Electronic Science and Engineering, 12466 Xiamen University, Xiamen 361005, China; Institute of Electronics, National Yang Ming Chiao Tung University, Hsinchu 300, Taiwan; 12466 Shenzhen Research Institute of Xiamen University, Shenzhen 518057, China

**Keywords:** terahertz sensing, dielectric metasurfaces, Fano resonances, bound state in the continuum, biomolecular detection, chiral sensing

## Abstract

Terahertz (THz) technology has attracted significant global interest, particularly in sensing applications, due to its nonionizing feature and sensitivity to weak interactions. Recently, owing to the advantages of low optical loss and the capability to support both electric and magnetic high-quality factor (high-Q) resonances, dielectric metasurfaces have emerged as a powerful platform for multiscenario terahertz sensing applications. This review summarizes recent advancements in dielectric metasurface-assisted THz sensing. We begin with an overview of the mechanisms and properties of dielectric metasurfaces with high-Q factors. Next, we discuss typical fabrication techniques for these terahertz dielectric metasurfaces. We then explore the diverse terahertz sensing applications across various scenarios, including biomolecule sensing, biomedical detection, environmental monitoring, and chiral sensing. Finally, we provide perspectives on the future development of this promising research field.

## 1 Introduction

Metasurfaces, composed of subwavelength artificial micro- or nanostructures arranged in specific patterns, enable unprecedented control over the amplitude, phase, and polarization of electromagnetic waves [1]. This capability has made them invaluable in a wide range of applications, particularly in the terahertz (THz) regime, where metasurfaces significantly enhance the interaction between light and matter. Terahertz technology, with its nonionizing properties and high sensitivity to weak molecular interactions, has drawn growing attention for its potential in fields such as medical diagnostics, environmental monitoring, molecular detection, and security screening.

Metasurfaces are generally categorized into metallic and dielectric types [2]. Metallic metasurfaces rely on plasmonic resonances, where free electron oscillations on the metal surface are highly responsive to environmental changes, making them suitable for various sensing applications [3]. Numerous studies have demonstrated the potential of plasmonic metasurfaces for THz sensing. For example, in 2017, Ulibarri et al. presented a cross-dipole metasurface sensor that functioned effectively under both normal and oblique incidence with high-quality factor (high-Q) of 0.94 and sensitivity of 180 GHz/RIU [4]. In 2018, Xie et al. achieved high-Q Fano resonance with a *Q* factor of 58 and sensitivity of 105 GHz/RIU using a four-metal resonant cavity structure [5]. Additionally, Liu et al. in 2020 observed giant asymmetric transmission and circular dichroism in chiral metamaterials, which can achieve chiral sensing with the chiral parameter *κ* up to 450 [6]. Zhang et al. in 2021 developed a terahertz biosensor capable of detecting glioma cells with high sensitivity of 496.01 GHz/RIU using an electromagnetically induced transparency (EIT) mechanism [7] and the mixed resonant mode metasurface with a sensitivity of 403.7 GHz/RIU developed by Zhang et al. [8]. However, despite these achievements, metallic metasurfaces face limitations such as high thermal conductivity, low melting points, and significant ohmic dissipation, leading to energy loss and low Q factors [9]. To address these challenges, dielectric metasurfaces have emerged as a more promising alternative, offering unique low-loss resonances and superior optical performance [10]. Compared to their metallic counterparts, dielectric metasurfaces exhibit lower optical losses and support higher Q factors, making them suitable for a wide range of terahertz applications, such as wavefront engineering [11], [12], polarization control [13], [14], [15], energy absorption [16], [17], [18], [19], cloaking [20], spatial imaging [21], and high-speed communication [22], [23]. Their ability to support both electric and magnetic resonances further enhances their versatility, especially for high-sensitivity THz sensing applications [24], [25] that require precise detection of subtle changes in refractive indices or molecular structures. Further advancements such as Wang et al. utilized an asymmetric tetramer cluster metasurface to achieve ultrahigh biosensing with *Q* factor above 10^5^ and sensitivity of 489 GHz/RIU through bound states in the continuum (BIC) resonances [26] and BIC-based metasurface with *Q* factor of 69,238 and high sensitivity of 535 GHz/RIU designed by Li et al. [27], which are much higher than the metalic metasensor in terms of Q factor and sensitivity, showcased the potential of dielectric metasurfaces.

**Figure 1:**
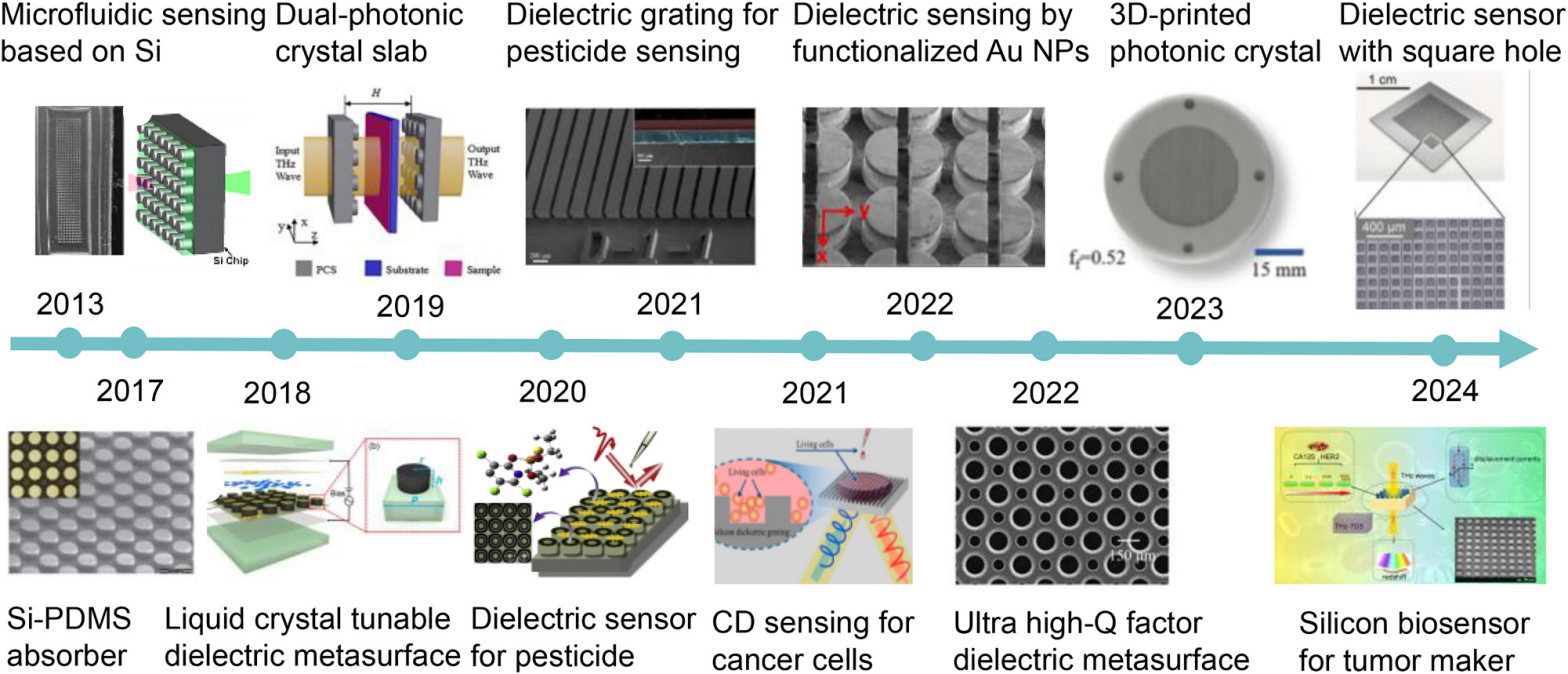
Some significant achievements of terahertz dielectric metasurfaces, especially in the field of biosensing, from 2013 to the end of 2024. Data derived from reported studies [28], [29], [30], [31], [32], [33], [34], [35], [36], [37], [38], [39]. Reproduced with permission [28], [29], [30], [31], [32], [33], [34], [35], [36], [37], [38], [39].

Recent advancements in dielectric metasurfaces have demonstrated their remarkable capabilities in various terahertz sensing applications, particularly in the biosensing domain. As illustrated in Figure 1, significant progress has been made from 2013 to 2024, with dielectric metasurfaces transitioning into highly efficient and sensitive sensing platforms. One of the early breakthroughs occurred in 2013 when Fan et al. fabricated a photonic crystal (PhC) based on silicon pillars for microfluidic sensing in the THz band [28]. While this achievement highlighted the promise of dielectric metasurfaces, it also revealed fabrication challenges, particularly on low refractive index substrates. In 2017, Liu et al. developed a dielectric metasurface absorber using micron-sized silicon discs fabricated on boron-doped silicon-on-insulator (SOI), which were flipped after wet etching the oxide layer and integrated onto a thick polydimethylsiloxane (PDMS) substrate, achieving a maximum absorption peak of 97.5 % [29]. However, the static nature of the resonance in these metasurfaces remained a limitation in some specific applications. To enhance tunability, Zhou et al. integrated liquid crystal into dielectric metasurfaces, enabling dynamic THz absorption modulation and tunable sensing applications through external stimuli [30]. Other notable designs include silicon-based photonic crystal cavities with a high *Q* factor of 529 and a 31-fold enhancement in selectivity for lactose detection [31] as well as highly doped silicon metasurfaces with broadband and multiband absorption for enhanced trace pesticide chlorpyrifos sensing [32], [33]. Beyond these advances, dielectric metasurfaces have also shown promise in circular dichroism (CD) sensing, providing enhanced polarization responses and improved detection of chiral molecules. For example, Zhang et al. designed a silicon subwavelength grating and reflective THz time-domain CD sensing system capable of quantitatively detecting cancer cells in liquids with high sensitivity, achieving a detection limit of 10^4^ cells/mL [34].

In recent years, dielectric metasurfaces have been employed in a broader range of THz sensing in various scenarios, including trace pesticide sensing, cell detection, immunosensing of small molecules, and cancer diagnostics. For instance, a semicircular dielectric metasurface with specific immunobinding of human influenza hemagglutinin tag protein (HA antigen), achieving HA antigen detection at nanomolar concentrations of 1.05 nmol/mL [35]. With the growing demand for sensors with higher Q factors, significant efforts have been devoted to exploring BIC, a promising approach for achieving ultra-high-Q resonances in metasurfaces. In 2022, Wang et al. developed an asymmetric silicon circular hole metasurface with an experimentally measured Q factor of 1,049, tripling the previous record [36]. Furthermore, Pilozzi et al. demonstrated a 3D-printed one-dimensional photonic crystal slab capable of transitioning from Fano into high-Q BIC resonances, with a *Q* factor reaching 33 [37]. Additionally, Lin et al. further developed an all-silicon square hole meta-sensor with a figure of merit (FOM) of 15.2, enabling proline detection through multiple high-Q resonances driven by BIC [38]. Recently, the practical applications of THz dielectric metasurface biosensors have expanded from laboratory research into bioengineering applications. For instance, a silicon pillar array-based biosensor demonstrated high sensitivity in detecting tumor markers with a detection limit of 0.1 ng/mL, offering a promising platform for early cancer diagnostics [39]. These advancements indicate the growing potential of dielectric metasurfaces in THz sensing, particularly in biomedical applications, where their precision and sensitivity are poised to make significant contributions.

In this review, we aim to provide a comprehensive analysis of recent progress in dielectric metasurface-assisted terahertz sensing. First, we explore the underlying physical mechanisms that enable dielectric metasurfaces to achieve high-Q resonances, such as Fano resonance, BIC, EIT, and Mie resonance. We then discuss state-of-the-art fabrication techniques that are driving advancements in metasurface performance. Finally, we delve into the practical applications of dielectric metasurfaces in multiple sensing scenarios, including biomolecule sensing, biomedical detection, environmental monitoring, and chiral sensing. We also outline potential future research directions, where dielectric metasurfaces could play a transformative role in advancing terahertz technology.

## 2 The mechanisms of dielectric metasurfaces with high-Q factors

Dielectric metasurfaces are capable of supporting high-Q resonances through a variety of extraordinary physical mechanisms, including Fano resonances, BIC, EIT, and Mie resonances. By leveraging these mechanisms, metasurfaces exhibit sharp spectral features and high sensitivity, making them ideal for high-precision sensing applications. This section delves into these fundamental mechanisms that enable high-Q resonances in dielectric metasurfaces.

### 2.1 Fano resonance

**Figure 2:**
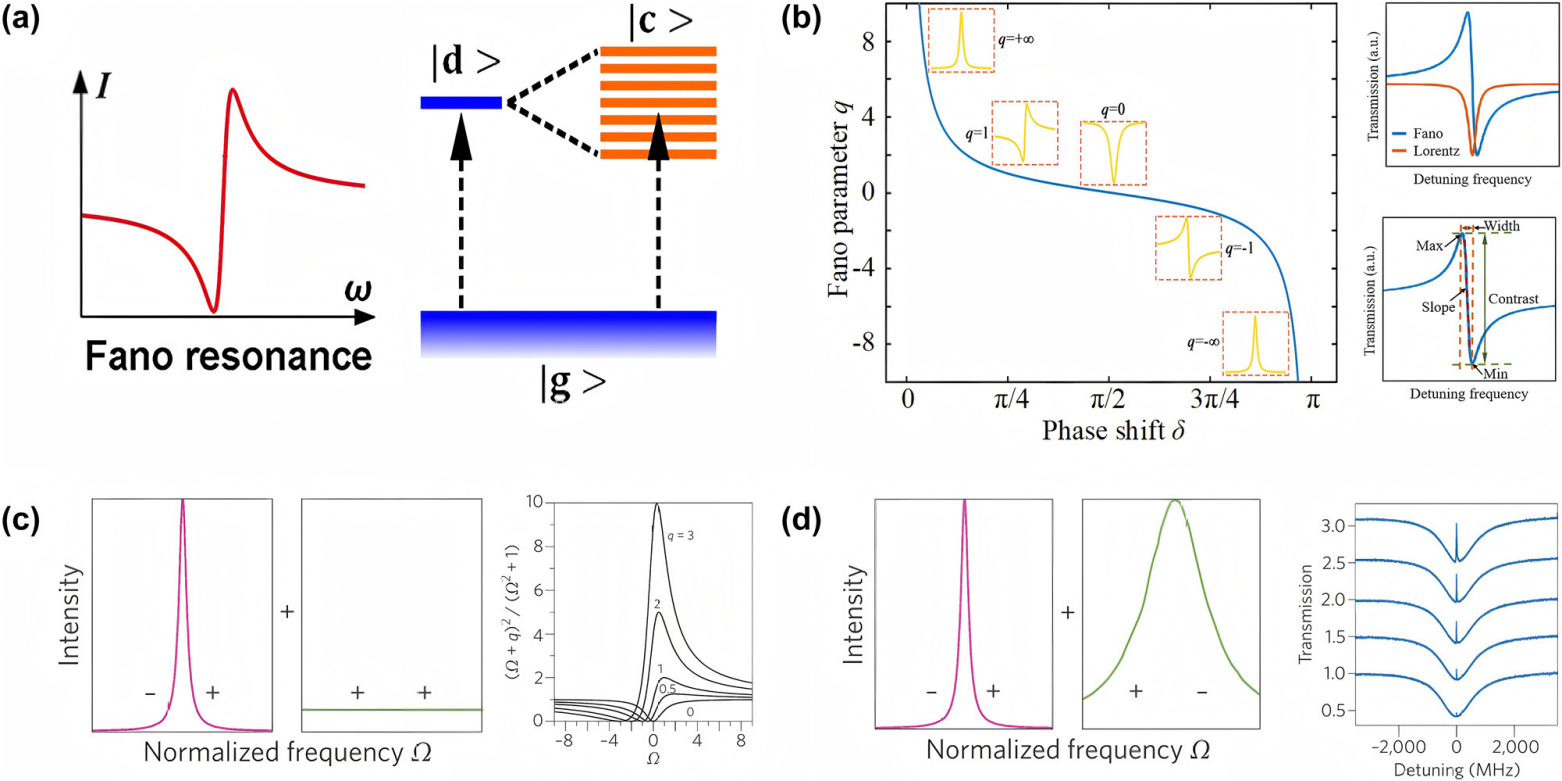
Schematic illustration of the Fano resonance. (a) Fano resonance with an asymmetric line shape [42]. (b) Fano parameter versus phase shift and the Fano response function [43], [44]. (c) Spectral shapes of Fano resonance versus *q* values [43]. (d) Schematic of EIT and its relationship between spectral shape and detuning values [43]. (a) Reproduced with permission. Copyright 2017, Nature Publishing Group. (b)–(d) Reproduced with permission. Copyright 2017, Nature Publishing Group.

Fano resonance has garnered significant attention in recent years due to its potential to enhance the sensitivity and detection limits of sensors. This is primarily because of the abrupt changes in both phase and amplitude that occur during resonance, making it ideal for applications requiring sharp spectral features. Fano resonance can be induced in dielectric metasurfaces by carefully tailoring the geometry of the meta-atoms [40], [41]. As illustrated in Figure 2(a), Fano resonance arises from the interference between a discrete localized state and a continuum of states, producing a unique resonance response characterized by sharp spectral features, narrow linewidths, and high-*Q* factors [42]. Fano resonance occurs when a discrete state interferes with a continuum, resulting in a distinctive asymmetric line shape. This effect is commonly observed in absorption spectra *σ*(*E*) and can be described by the well-known Fano formula [43]:

(1) $$\sigma \left(E\right)=D^{2}\frac{{\left(q+\Omega \right)}^{2}}{1+\Omega^{2}}$$

(2) $$\;\Omega =\frac{2\left(E-E_{0}\right)}{G}$$

(3) $$\;D^{2}=4\sin^{2}\delta$$

where *E* is the energy, *q* is the Fano parameter that characterizes the degree of asymmetry of the line shape, *δ* is the phase shift of the continuum relative to the discrete state, Ω represents the reduced energy, *E*_0_ is resonance energy, and *G* is the resonance width, respectively. The parameter *q*, defined as *q* = cot(*δ*), governs the degree of interference between the discrete and continuum states, allowing for the fine-tuning of the resonance profile.

Fano resonances can be observed not only in absorption spectra but also in transmission and scattering spectra in various systems. Depending on the interference profile, resonance behavior can be classified into two types: Lorentz and Fano line shapes. Lorentz resonances exhibit symmetric profiles, while Fano resonances display a sharp, asymmetric line shape with steep slopes. Under certain conditions, the transition between these two line shapes can occur.

As shown in Figure 2(b), the key Fano parameter *q*, which characterizes the asymmetry of the resonance, is determined by the phase shift *δ* between the discrete and continuum states [43], [44]. When |*q*| → ∞, the discrete state dominates, leading to a symmetric Lorentz line shape, which represents a special case of the Fano resonance. On the other hand, when *q* = 0, the continuum state dominates, resulting in a quasi-Lorentz antiresonance. When *q* = 1, the two states interfere destructively, producing a resonance where the transition intensities of both states are equal.

The value of *q* can be manipulated by adjusting the geometric and material parameters of the metasurface, as depicted in Figure 2(c). This tunability allows for a wide range of resonance behaviors, making Fano resonances highly versatile for different sensing applications. Additionally, Figure 2(d) compares the coupling scenarios of Fano resonance and EIT, helping to differentiate between the two resonance mechanisms and understand their unique properties.

### 2.2 Bound states in the continuum

**Figure 3:**
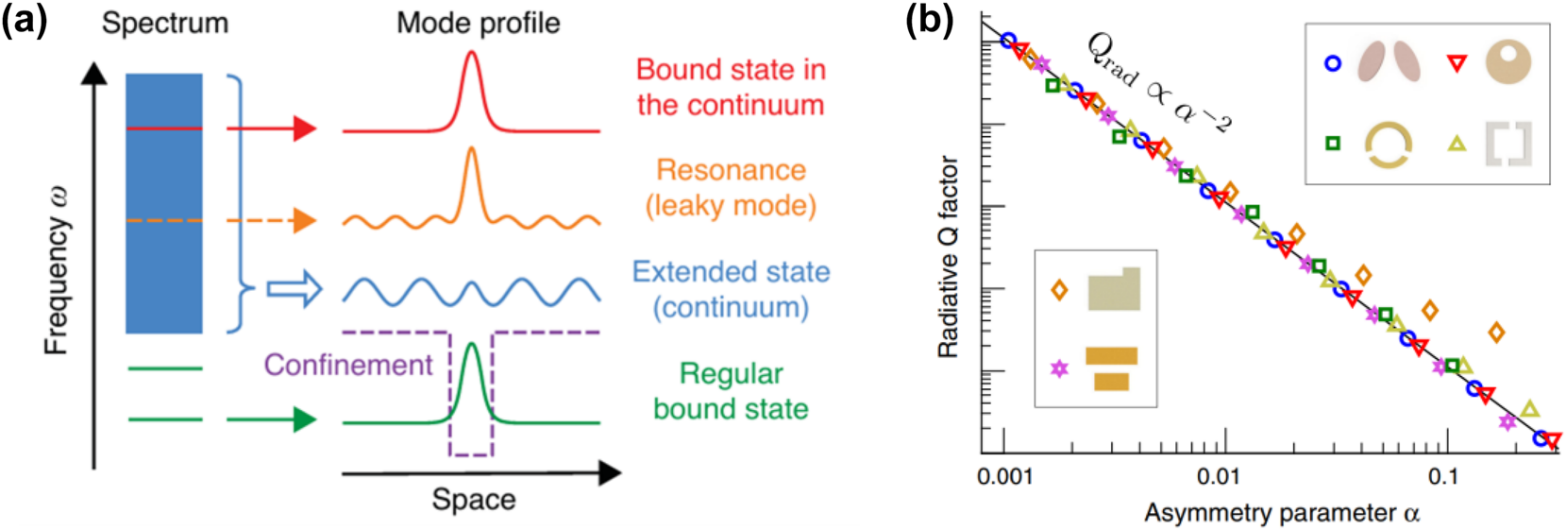
Symmetry-protected BICs. (a) Illustration of a BIC [45]. (b) Dependence of the *Q* factor of the metasurfaces with symmetry-broken meta-atoms on the asymmetry parameter *α*, following *Q* = *Q*_0_ *α* ^−2^ [46]. (a) Reproduced with permission. Copyright 2016, Nature Publishing Group. (b) Reproduced with permission. Copyright 2018, APS Publishing Group.

In recent years, the concept of BIC has been introduced into optical metasurfaces, as illustrated in Figure 3(a) [45]. An ideal BIC is characterized by a spectral feature with a vanished linewidth and an infinitely high radiation Q factor, representing a strong interaction between light and matter in a periodic system without coupling to free-space radiation. BICs can be classified into two main types based on their formation mechanisms: symmetry-protected BICs and accidental BICs. This section reviews the most recent progress on these two types of BICs supported by all-dielectric metasurfaces.

#### 2.2.1 Symmetry-protected BICs

Symmetry-protected BICs are easy to access, even in simple systems, by tailoring or combining meta-atoms. As long as the subwavelength structure exhibits *C*_2_ symmetry, modes of different symmetry classes can decouple from the far-field. In these cases, the electromagnetic field is constrained within a resonance mode of even symmetry, which remains orthogonal to the odd-symmetric radiative mode. As a result, external excitation cannot drive the mode, and it becomes what is known as a symmetry-protected BICs [47], [48].

In a two-dimensional periodic metasurface with symmetry, a special point exists in wavevector space, known as the Γ point, where leaky modes have no horizontal component, and the parallel component exhibits 180° rotational symmetry. At the Γ point, the even-symmetric mode is completely confined within the structure due to *C*_2_ symmetry, forming a symmetry-protected BIC, while the odd-symmetric mode radiates out of the structure. As the system moves away from the Γ point, the horizontal component of the leaky modes becomes nonzero, and the parallel component loses its invariance. As symmetry protection is lost, the BIC couples with radiation modes, forming a quasi-BIC (QBIC).

QBICs can be easily accessed by breaking the *C*_2_ symmetry of the structures. Common structures that generate QBICs include a pair of inclined double ellipses at specific angles, split rings or rectangular rings of varying sizes or positions, rectangular strips of different lengths, or tetramer clusters composed of four cylinders with varying positional deviations, as illustrated in Figure 3(b). The interaction with far-field radiation results in QBICs exhibiting nonzero resonance linewidths in the spectrum due to resonance damping.

A key physical parameter describing the resonance strength of QBICs is given by the dimensionless ratio *Q* = *ω*_0_∕2*γ*, where *ω*_0_ is the resonance frequency, and *γ* is the resonance damping rate. The Q factor of symmetry-protected BICs is directly related to the degree of symmetry breaking in the structure. This relationship can be described by the expression *Q* = *Q*_0_ *α* ^−2^, where *Q*_0_ is a constant determined by the structure, and *α* is the asymmetry coefficient. And *α* increases as symmetry is broken, as reported by Kivshar’s group [46]. As shown in Figure 3(b), the Q factor decreases as *α* increases, eventually decaying to the level of conventional resonances.

#### 2.2.2 Friedrich–Wintgen (FW) BIC

**Figure 4:**
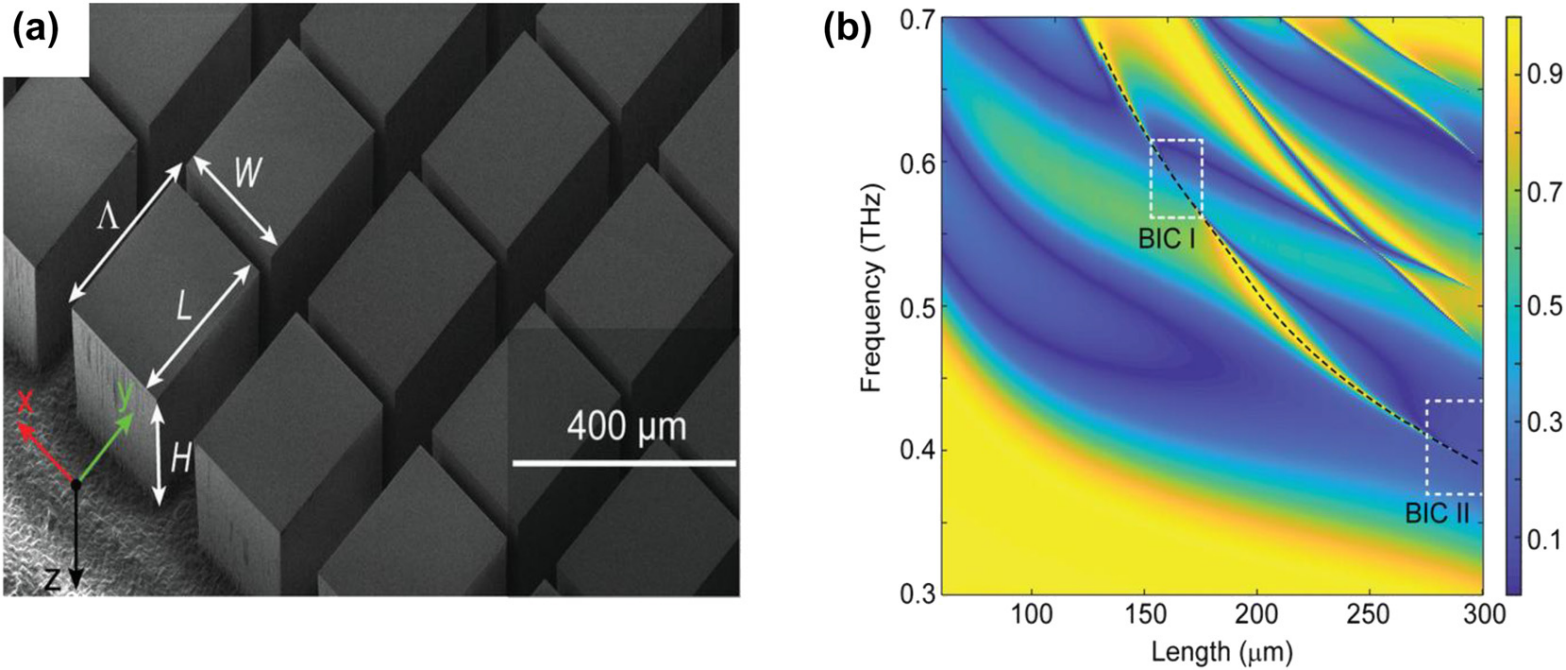
Dielectric metasurface with FW BIC. (a) Schematic of the dielectric metasurface at normal incidence. (b) Map of transmission spectra by sweeping the cuboid length, where the low-frequency mode is marked by a black dashed curve, with two BIC regions indicated on this mode [50]. (a) and (b) Reproduced with permission. Copyright 2019, Wiley Publishing Group.

In addition to symmetry-protected BICs, another class of accidental BICs arises from the interaction between different eigenstates. These accidental BICs, which can be avoided by geometrical or angular detuning, are common in photonic slabs. A well-known type within this category is the Friedrich–Wintgen (FW) BIC, as shown in Figure 4, which is prevalent in dielectric metamaterials [49], [50]. FW BICs occur due to phase cancellation interference between two radiation modes, which requires precise structural tuning. According to coupled-mode theory, when two resonances exist in the same cavity and are coupled to the same radiation channel, the amplitudes of these resonances evolve according to the following Hamiltonian model [51]:

(4) $$H=\left[\begin{array}{cc} \omega_{1} & \kappa \\ \kappa & \omega_{2} \end{array}\right]-i\left[\begin{array}{cc} \gamma_{1} & \sqrt{\gamma_{1}\gamma_{2}}\\ \sqrt{\gamma_{1}\gamma_{2}} & \gamma_{2} \end{array}\right]$$

where, *ω*_1_ and *ω*_2_ represent the resonant frequencies of the two resonant modes, *γ*_1_ and *γ*_2_ denote their respective radiative loss, and *κ* represents the coupling between the two resonant modes. When these two resonant modes are coupled to the same radiation channel, the coupling term $\sqrt{\gamma_{1}\gamma_{2}}$ generated by interference can be adjusted to produce a BIC. It is found that when $\kappa \left(\gamma_{1}-\gamma_{2}\right)=\left(\omega_{1}-\omega_{2}\right)\sqrt{\gamma_{1}\gamma_{2}}$ , one of the eigenvalues becomes purely real, the corresponding coupling mode becomes BIC, while the other mode remains radiative. This equation was first proposed by Friedrich and Wintgen, thus giving rise to the term FW BIC for this class of continuous domain bound states [49]. FW BICs occur near the point of frequency crossing (*ω*_1_ ≈ *ω*_2_) between the two uncoupled resonances, typically when *κ* ≈ 0, or *γ*_1_ ≈ *γ*_2_.

In general, FW BICs are observed when the number *N* of coupled modes exceeds the number *M* of radiation channels, although the complexity of tuning increases as *M* grows [52], [53]. This form of BIC was first introduced in studies of atomic and molecular potentials. In photonics, FW BICs have been realized in dielectric structures such as microwave waveguides and photonic crystal waveguides [54], [55], offering exciting possibilities for the control of light and enhancing Q factors in a variety of optical devices.

### 2.3 Electromagnetically induced transparency

**Figure 5:**
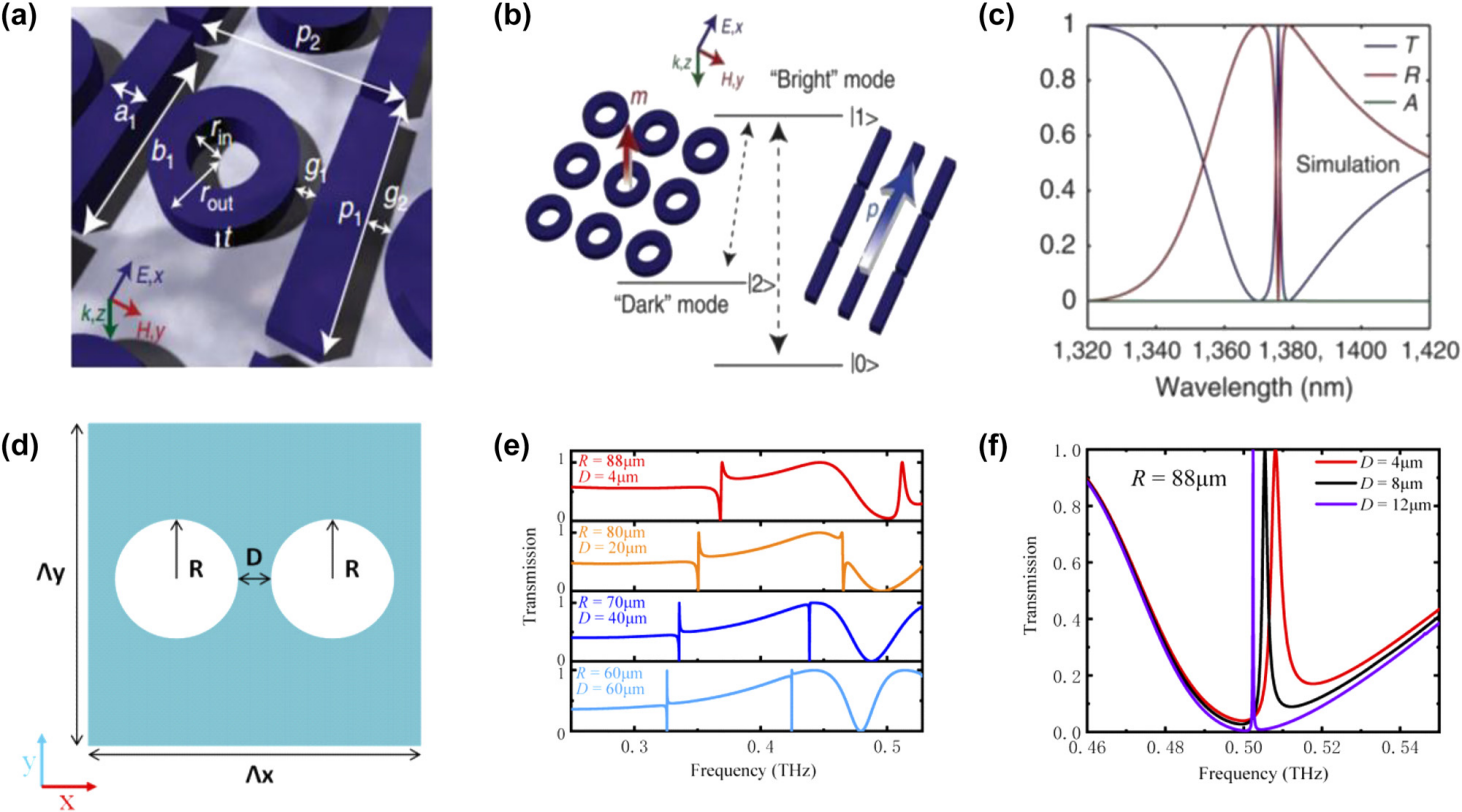
EIT dielectric metasurfaces. (a) Diagram of the metasurface with bright–dark mode coupling [61]. (b) Schematic of interference between the bright- and dark-mode resonators. (c) Simulated transmittance (blue curve), reflectance (red curve), and absorption (green curve) spectra of the metasurface. (d) Unit cell of the metasurface with bright–bright mode coupling [62]. (e) Transmission spectra of metasurface with different radii of the air holes. (f) Transmission transparency windows for different distances between the two air holes when *R* = 88 μm. (a)–(c) Reproduced with permission. Copyright 2014, Nature Publishing Group. (d)–(f) Reproduced with permission. Copyright 2021, Optica Publishing Group.

Terahertz metasurfaces offer unique ways to manipulate electromagnetic waves for various applications. In recent years, the concept of EIT has attracted wide interest for its potential applications in sensing [56], controllable delay lines, nonlinear effects, and slow-light equipment for its strong dispersion characteristic [57], [58]. EIT is a quantum phenomenon that results from the destructive interference between different excitation pathways in a three-level atomic system, making an initially opaque medium transparent to a probe laser beam [59]. To generate EIT analogs in metasurfaces, two primary approaches can be employed: (i) bright–dark mode coupling and (ii) bright–bright mode coupling. In the bright–dark mode coupling scenario, the bright mode resonator is characterized by a low Q factor and high radiation, while the dark mode exhibits a high Q factor and cannot be directly excited by the incident wave. This approach typically involves a bright mode resonator coupled directly to the incident wave, along with a dark mode activated by the bright mode. For effective EIT, the bright and dark resonances should have the same resonance frequency but have different linewidths, allowing for them to couple and produce EIT [60], [61]. Dielectric metasurfaces can enhance the performance of their plasmonic counterparts in mimicking EIT due to their reduced absorption loss [61]. Figure 5(a)–(c) present the results of a work of dielectric metasurface showing an EIT resonance in the near-infrared regime. As illustrated in Figure 5(a) and (b), the dielectric metasurface consists of a periodic lattice with a silicon rectangular bar and ring resonators. The collective oscillations of the bar resonators form the “bright” mode, while the ring resonators interacting through near-field coupling generate the “dark” mode by suppressing radiative loss.

The interference in the metasurface can be described by the coupled differential equations [61]:

(5) $${\dot{x}}_{1}-j\left(\omega_{0}+j\gamma_{1}\right)x_{1}+j\kappa x_{2}=gE_{0}e^{j\omega t}$$

(6) $${\dot{x}}_{2}-j\left(\omega_{0}+\delta +j\gamma_{2}\right)x_{2}+j\kappa x_{1}=0$$

where, *x*_1_ and *x*_2_ represent the magnitudes of the bright and dark mode resonances, respectively, and *γ*_1_ and *γ*_2_ are their losses. *ω*_0_ is the resonance frequency of the bright mode, *δ* is the detuning of resonance frequency, *g* is the bright-mode dipole-coupling strength to the incident electric field *E*_0_, and *κ* is the coupling coefficient between the two resonators. The simulated transmittance, reflectance, and absorption spectra of the designed structure, shown in Figure 5(c), display a distinct EIT-like peak at a wavelength of 1,376 nm. The transparency window peak approaches unity, making it possible to realize slow-light devices that are highly dispersive and lossless.

The bright–bright mode coupling approach involves frequency detuning and hybridization of two bright modes that are close to each other. It was demonstrated that a high Q factor analog of EIT using an all-silicon metasurface in the terahertz regime by coupling a toroidal dipole high-Q Fano resonance with a low-Q magnetic dipole mode [62], as illustrated in Figure 5(d). This EIT can be easily adjusted by varying the distance between the air holes. When the radius *R* is increased to 88 µm, the detuning between the two resonances becomes minimal, leading to destructive interference (bright–bright mode coupling) and resulting in a high-Q EIT-like resonance at approximately 0.5 THz, as depicted in Figure 5(e) and (f). The flexibility of bright–dark and bright–bright mode coupling mechanisms provides multiple pathways to design high-Q EIT-like resonances. With the ability to tailor the interaction between resonators through structural tuning, EIT-based metasurfaces hold great potential for future developments in high-precision terahertz sensing and optical devices.

### 2.4 Mie resonance and multipole decomposition

Mie resonance refers to the strong absorption and scattering phenomenon that occurs when an optical field interacts with nanoparticles. While Maxwell’s equations can theoretically explain all light–matter interactions, finding exact analytical solutions becomes complex for nanoparticles that deviate from spherical shapes. In 1908, Gustav Mie provided an exact solution to Maxwell’s equations to explain the light scattering and absorption of gold nanorods in colloidal solutions, a development that laid the foundation for the subsequent Mie theory. This theory describes the scattering of light by spherical particles at subwavelength scales, which is a fundamental aspect of classical electrodynamics [63].

For a single isolated dielectric sphere with radius *r* and refractive index *n*, the scattered field can be decomposed into a series of multipole resonances. The 2*m*-pole term of the scattered electric field is proportional to:

(7) $$a_{m}=\frac{n\psi_{m}\left(nx\right)\psi_{m}^{\prime }\left(x\right)-\psi_{m}\left(x\right)\psi_{m}^{\prime }\left(nx\right)}{n\psi_{m}\left(nx\right)\xi_{m}^{\prime }\left(x\right)-\psi_{m}^{\prime }\left(nx\right)\xi_{m}\left(x\right)}$$

and the 2*m*-pole term of the scattered magnetic field is proportional to:

(8) $$b_{m}=\frac{\psi_{m}\left(nx\right)\psi_{m}^{\prime }\left(x\right)-n\psi_{m}\left(x\right)\psi_{m}^{\prime }\left(nx\right)}{\psi_{m}\left(nx\right)\xi_{m}^{\prime }\left(x\right)-n\psi_{m}^{\prime }\left(nx\right)\xi_{m}\left(x\right)}$$

where, *ψ*_*m*_(*x*) and *ξ*_*m*_(*x*) are Riccati–Bessel functions, *x* = *k*_0_ *r*, and *k*_0_ denotes the wave number of the electromagnetic wave in vacuum, and *a*_*m*_ and *b*_*m*_ correspond to the scattering coefficients of the electric and magnetic responses of the spherical particles of the medium, respectively. The total scattering cross section of a spherical particle can then be expressed by the sum of these two scattering coefficients:

(9) $$C_{\mathrm{sca}}=\frac{2\pi }{k^{2}}\overset{\infty }{\underset{m=1}{\sum }}\left(2m+1\right)\left({\left|a_{m}\right|}^{2}+{\left|b_{m}\right|}^{2}\right)$$

From (9), the scattering characteristics can be engineered for specific applications by adjusting the geometrical parameters of the nanoparticles.

Mie scattering in dielectric resonators involves contributions from different multipole resonances, such as electric dipole (*P*), magnetic dipole (*M*), electric quadrupole (*Q*_*e*_), and magnetic quadrupole (*Q*_*m*_) resonances, which affect both the near-field and far-field behavior of electromagnetic waves [64]. Multipole decomposition is an important method for analyzing electromagnetic scattering and Mie resonance. It decomposes complex electromagnetic fields into multiple independent modes with a series of Taylor expansions in Cartesian coordinates. The scattering section is calculated by [65]:

(10) $$\begin{array}{ll} P_{\mathrm{scat}} & =\frac{k_{0}^{4}\sqrt{\varepsilon_{d}}}{12\pi \varepsilon_{0}^{2}c\mu_{0}}{\left|p_{i}+\frac{ik}{c}T_{i}\right|}^{2}+\frac{k_{0}^{4}\varepsilon_{d}\sqrt{\varepsilon_{d}}}{12\pi \varepsilon_{0}c}{\left|m_{i}\right|}^{2}\\ & \;+\frac{k_{0}^{6}\varepsilon_{d}\sqrt{\varepsilon_{d}}}{160\pi \varepsilon_{0}^{2}c\mu_{0}}{\left|Q_{ij}^{\left(e\right)}\right|}^{2}+\frac{k_{0}^{6}\varepsilon_{d}\sqrt{\varepsilon_{d}}}{160\pi \varepsilon_{0}c}{\left|Q_{ij}^{\left(m\right)}\right|}^{2} \end{array}$$

where, the contributions from *P*, *M*, *Q*_*e*_, *Q*_*m*_, and toroidal dipole (*T*) moments are expressed as:

(11) $$p_{i}=i/\omega \int J_{i}dv$$

(12) $$m_{i}=1/2\int {\left(r\times J\right)}_{i}dv\;$$

(13) $$Q_{ij}^{\left(e\right)}=i/\omega \int r_{j}J_{i}+r_{i}J_{j}-2/3*\delta_{ij}\left(r\cdot J\right)dv$$

(14) $$Q_{ij}^{\left(m\right)}=1/3\int \left({\left(r\times J\right)}_{i}r_{j}\right)+\left({\left(r\times J\right)}_{j}r_{i}\right)dv\;$$

(15) $$T_{i}^{\left(e\right)}=1/10\int \left(J\cdot r\right)r_{i}-2r^{2}J_{i}dv\;$$

**Figure 6:**
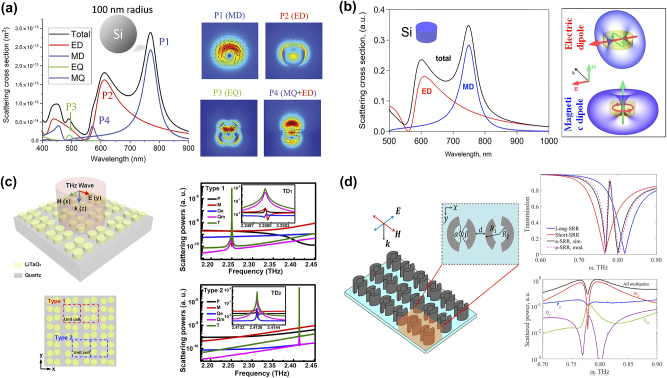
Mie resonances and multipole decompositions for various dielectric metasurfaces. (a) A 100-nm-radius silicon nanosphere and its electric field distributions [66]. (b) A silicon nanodisk and its multipole scattering spectra and radiation patterns [67]. (c) Asymmetric metasurface and its multipole scattering spectra [26]**.** (d) Asymmetric split ring metasurface and its transmission and multipole scattering spectra [68]. (a) Reproduced with permission. Copyright 2019, De Gruyter Publishing Group. (b) Reproduced with permission. Copyright 2017, ACS Publishing Group. (c) Reproduced with permission. Copyright 2020, De Gruyter Publishing Group. (d) Reproduced with permission. Copyright 2019, Optica Publishing Group.

For high refractive index spherical particles, as shown in Figure 6(a), the scattering cross section consists of a series of sharp resonance peaks, which are excited by different electric and magnetic resonance modes [66]. Beyond spherical or cubic structures, recent advancements have expanded the geometric diversity of dielectric metasurfaces to include rings, disks, and rods. For example, in 2017, Kruk et al. designed a silicon nanodisk with a thickness of 260 nm and a radius of 200 nm, exhibiting distinct Mie resonance peaks from both magnetic dipole and electric dipole resonances, as shown in Figure 6(b) [67]. Calculating the coefficients of these resonances reveals that both electric and magnetic fields are confined within the dielectric particle. For high-index dielectric nanoparticles, when the wavelength inside the particle is comparable to its diameter, 2*r* ≈ *λ*/*n*, where *n* is the refractive index of the particle material, *r* is the nanoparticle radius, and *λ* is the wavelength of light, the lowest-order Mie resonance is a magnetic dipole, originating from the magnetic field perpendicular to the toroidal displacement current within the particle. The second lowest-order resonance is an electric dipole, which enhances the displacement current inside the particle and generates a strong toroidal magnetic field around the particle. In small dielectric nanoparticles, these magnetic and electric dipole resonances dominate the scattering spectrum. However, as the size of the particles increases, higher-order resonances such as magnetic and electric quadrupoles begin to contribute significantly to the scattering properties.

In another study, Wang et al. demonstrated a dielectric metasurface with asymmetric tetramer clusters [26], as shown in Figure 6(c). Introducing asymmetry in the relative positions of two dielectric cylinders excites both intracluster and intercluster TD resonances, producing QBIC resonances with an ultra-high Q factor of 1.2 × 10^5^ and a maximum sensitivity of 489 GHz/RIU for trace analyte detection. Multipole decomposition was used to analyze the contributions of *P*, *M*, *Q*_*e*_, *Q*_*m*_, and *T*, as shown in Figure 6(c). For resonance 1 at 2.25 THz and resonance 2 at 2.42 THz, toroidal dipole contribution dominates, followed by *Q*_*m*_, while *P*, *M*, and *Q*_*e*_ are suppressed. This decomposition highlights the contributions of intracluster (TD1) and intercluster (TD2) interactions. Furthermore, Ma et al. proposed a dielectric metasurface based on asymmetric split-ring resonators [68], as shown in Figure 6(d). It exhibited EIT excited by the length difference between the two arcs. The EIT transparency window is at around 0.78 THz with a Q factor of 75.7, indicating its potential for high-sensitivity sensing applications. Theoretical analysis showed that the EIT effect arose from the hybridization of two bright modes excited by magnetic dipoles. The far-field multipole scattering electromagnetic power based on the displacement current density revealed that magnetic dipole excitation dominated the two transmission dips, while other multipoles like *P*, *Q*_*e*_, and *Q*_*m*_ contributed to the broadening of the EIT peak, decreasing the Q factor due to radiation loss.

Mie theory, combined with multipole decomposition, provides a powerful framework for analyzing and controlling the scattering and resonance in dielectric metasurfaces. Multipole decomposition allows complex electromagnetic fields to be broken down into their constituent modes, enabling a deeper understanding of how light interacts with dielectric structures. The ability to tune multipole resonances through geometrical parameters opens up new possibilities for THz metasurfaces. In THz sensing, Mie resonances enable highly sensitive detection by amplifying the interaction between light and matter. As metasurfaces evolve beyond simple spherical or cubic shapes, the potential for Mie-based resonances in THz sensing will drive new opportunities for enhanced sensing platforms with high precision and sensitivity.

## 3 Fabrication

Compared to traditional THz metallic metasurfaces, which are typically fabricated using photolithography, metal deposition, and lift-off process, the manufacturing techniques for dielectric metasurfaces are more diverse, including conventional photolithography and dry etching [69], [70], self-assembled technology, and laser direct writing [71], [72], [73]. In this section, we will introduce the three primary fabrication methods for THz dielectric metasurface, to explore flexibility and diversity in fabrication.

### 3.1 Photolithography and etching

Silicon-based dielectric metasurfaces are typically fabricated using conventional UV lithography and dry etching processes. The fabrication begins with standard silicon wafer cleaning to remove contaminants, followed by spin-coating a photoresist layer onto the silicon substrate. The pattern is transferred to an etch mask layer via a photomask, and reactant gases such as SF_6_ or CF_4_ are used for dry etching, producing high-precision silicon pillars with vertical sidewalls. The remaining photoresist is then removed using acetone solvent or plasma cleaning.

For terahertz dielectric metasurfaces, the meta-atom dimensions typically range from tens to hundreds of micrometers, which can be achieved through UV lithography. The thicknesses of these meta-atoms, produced by dry etching, are significantly greater than the nanoscale thickness of optical dielectric metasurfaces fabricated by inductively coupled plasma (ICP) techniques. Deep reactive ion etching (DRIE), particularly the Bosch processes, is frequently employed to etch these structures with high aspect ratios. The Bosch process alternates between etching and deposition cycles using sulfur hexafluoride (SF_6_) for etching and octafluorocyclobutane (C_4_F_8_) for deposition. This method ensures anisotropic etching with high precision and vertical sidewalls. The Bosch process can achieve aspect ratios as high as 20:1, making it highly suitable for fabricating terahertz dielectric metasurfaces with accurate dimensions and well-defined vertical profiles.

**Figure 7:**
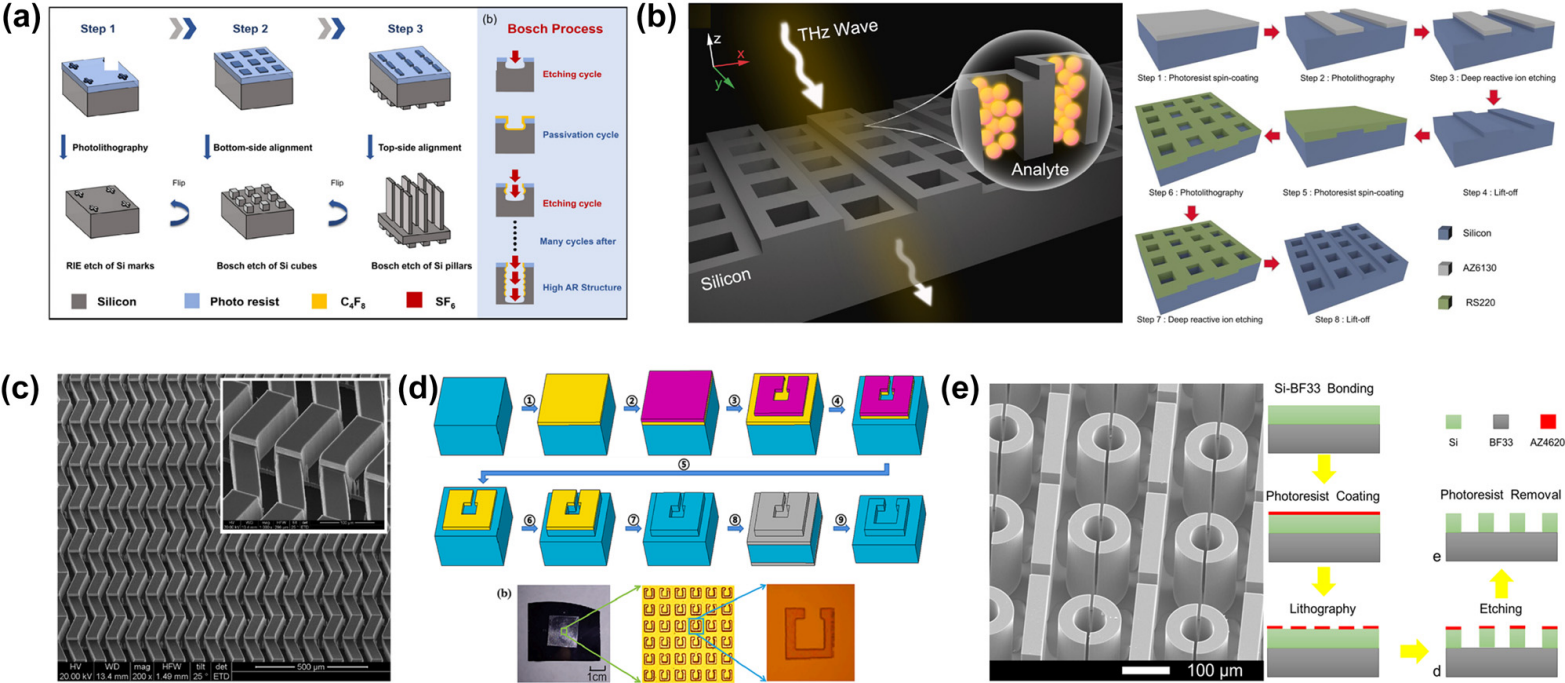
Fabrication process of dielectric metasurfaces. (a) A double-sided silicon metasurface using the Bosch process [74]. (b) A height asymmetry silicon hole metasurface [38]. (c) The SEM image of a height asymmetry metasurface [75]. (d) Fabrication process of metal hard mask etching [76]. (e) The SEM image of dielectric metasurface with a glass substrate and its fabrication flow [69]. (a) Reproduced with permission. Copyright 2023, Applied Sciences Publishing Group. (b) Reproduced with permission. Copyright 2024, Elsevier Publishing Group. (c) Reproduced with permission. Copyright 2019, Wiley Publishing Group. (d) Reproduced with permission. Copyright 2023, Elsevier Publishing Group. (e) Reproduced with permission. Copyright 2024, Micromachines Publishing Group.

Several recent studies highlight the versatility of the DIRE using Bosch process in fabricating dielectric metasurfaces, including double-sided dielectric metasurfaces and asymmetric dielectric metasurfaces with varying meta-atoms heights within one unit cell. For example, Yao et al. explored an optimized Bosch process to fabricate a double-sided silicon metasurface with a high aspect ratio (AR) of approximately 12:1, achieving excellent transmission properties that made the structure an efficient half-wave plate, as shown in Figure 7(a) [74]. They demonstrated how the etching and passivation influence sidewall verticality and etch depth. The fabrication involved etching silicon marks on the top surface, silicon cubes on the bottom, and etching silicon micropillars on the top surface. They employed a multistep etching process with gradient variations to mitigate the negative effects of accumulated reaction products, adjusting the cycle times and platen power to maintain the balance required for high aspect ratio silicon etching.

Lin et al. [38] explored asymmetric dielectric metasurfaces with broken symmetry BIC, showing high-Q factor resonances. They fabricated the asymmetric dielectric metasurface with varying etch heights along the *z*-axis by using dual photolithography and deep silicon etching to integrate stepped and square holes into a single meta-atom, as shown in Figure 7(b). In the first photolithography step, a strip pattern was transferred using AZ6130 photoresist, and etching created a height difference of 20 µm. Due to the nonplanar stepped substrate, the second photolithography employed spray coating with RS220 photoresist to ensure uniform thickness in grooves and on sidewalls. Subsequent etching formed through silicon vias. The height asymmetry of the metasurface excited QBIC modes, resulting in multiple high-Q factor transmission resonances, which are potentially useful for sensing applications.

In another work, dual photolithography was also utilized to fabricate height-asymmetric silicon dielectric metasurfaces, as shown in Figure 7(c) [75]. In that design, SU8-2050, a high-solid-content negative photoresist (50 μm), was spin-coated onto the unevenly etched wafer rather than spray coating, to minimize coating nonuniformity. Although thick photoresists are needed to compensate for substrate steps, they also limit the minimum line width. Cleaning steps, such as piranha solution (H_2_SO_4_:H_2_O_2_ mixture) and plasma etching of O_2_, are necessary to remove the highly cross-linked photoresist SU8-2050.

While photoresists like SU8, AZ4620, and SPR220 are commonly used as masking layers in DIRE, metal hard masks such as chromium, gold, or aluminum offer better etch resistance in deep or high-temperature etching processes. Yang et al. sputtered a copper layer onto the silicon surface and used wet etching to transfer it as a hard mask for square C-shaped etching, as shown in Figure 7(d) [76]. After the initial 20 µm etch, the copper mask was removed, and the silicon was further etched to a depth of 100 µm.

For THz dielectric metasurface sensors, a key factor in achieving a high-*Q* factor is the dielectric constant contrast between the dielectric resonators and the substrates. Although high dielectric materials enhance metasurface performance, they also introduce fabrication challenges, particularly when deposited on low refractive index substrates. From a technical standpoint, the dielectric resonators can be fabricated by directly growing high refractive index material layers on specific substrates using plasma-enhanced chemical vapor deposition (PECVD), followed by dielectric etching. However, for THz devices, where pattern height ranges from tens to hundreds of micrometers, deposition rates are often low and growing thick dielectric layers is time-consuming. To overcome these limitations, wafer-level anodic bonding techniques have been employed to bond the existing high-dielectric-constant silicon wafers with low-dielectric-constant borosilicate glass. Men et al. employed wafer-scale anodic bonding technology to combine existing silicon wafers with borosilicate glass. In this process, sufficiently high voltage and elevated temperature are applied to induce the migration of sodium ions in the glass, forming strong Si–O–Si chemical bonds between 300 µm thick BF33 glass and 200 µm thick silicon layer [69]. As illustrated in Figure 7(e), the silicon device layer is then etched using the Bosch process, achieving a deep-to-width ratio of 25:1.

Furthermore, in many previous dielectric metasurface fabrication, silicon is commonly used as both the dielectric resonator layer and the substrate [70]. It simplifies the fabrication process, although leading to power loss at the air–silicon interface. Therefore, the balance between fabrication complexity and resonance performance should be considered in the design.

### 3.2 Self-assembly techniques

In addition to conventional photolithography and etching techniques, chemical synthesis of spherical high-refractive-index micron-sized meta-atoms has also been explored. These spherical particles can be arranged via self-assembly, either on flexible substrates or within low-loss liquid polymers. Self-assembly techniques have emerged as an efficient method for fabricating terahertz metasurfaces with high-dielectric constant microscale spheres [77], [78]. By controlling self-assembly conditions, these microspheres can form ordered or disordered structures, realizing specific electromagnetic responses. Common materials used in this process include titanium dioxide, zirconium dioxide, and aluminum oxide, which are chemically synthesized and transferred onto adhesive substrates. This approach offers new insights into the design and manufacturing of dielectric metasurfaces. Compared to traditional photolithography and etching methods, self-assembly techniques are cost-effective and simpler, allowing for large-area manufacturing. Here, polymers like PDMS that exhibit weak absorption in the terahertz range are often served as the supporting substrates. Various composite materials, such as tunable materials, can be mixed into PDMS to create multifunctional inks, enhancing modulation capability. However, self-assembly is more suited to simple periodic arrangements, because its effectiveness is limited by template designs. Integrating self-assembly with other fabrication technologies, such as 3D printing, enables more complex arrangements.

**Figure 8:**
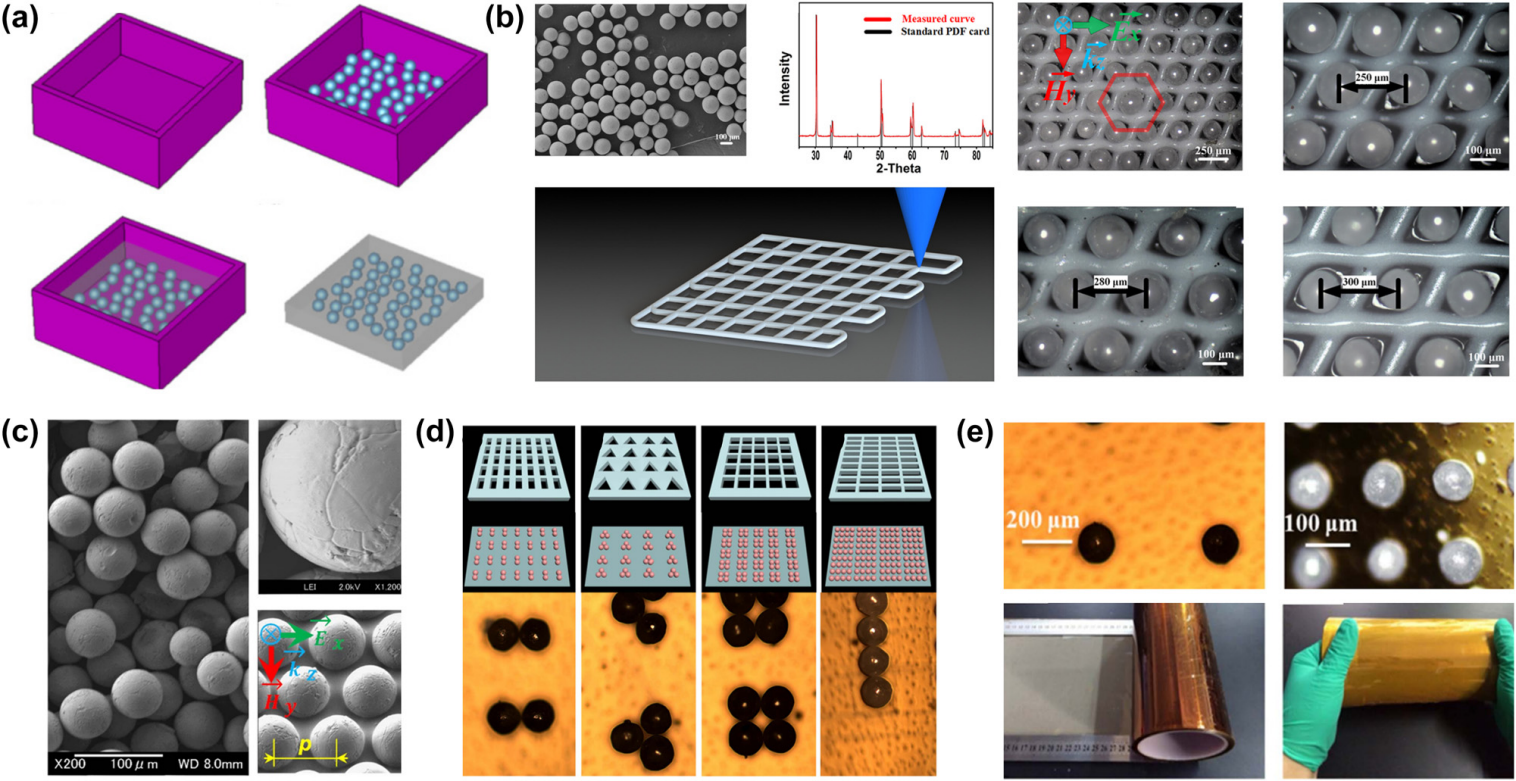
Self-assembly techniques for dielectric metasurfaces. (a) The fabrication process of dielectric microspheres in PDMS [80]. (b) Photograph of ZrO_2_ microspheres using a 3D direct-writing grid [81]. (c) Schematic diagram of the template and the image of different self-assembly methods [82]. (d) The SEM image of microspheres with hexagonal template [83]. (e) Photograph of large-area self-assembly [83]. (a) Reproduced with permission. Copyright 2018, Optica Publishing Group. (b) Reproduced with permission. Copyright 2015, Optica Publishing Group. (c) Reproduced with permission. Copyright 2019, Optica Publishing Group. (d)–(e) Reproduced with permission. Copyright 2022, Optica Publishing Group.

Currently, two primary methods are employed for self-assembly, including microtemplate-assisted self-assembly (MTAS), where microspheres are pushed into template holes to form a regularly ordered metasurface, and mixing microspheres into a medium such as PDMS. For example, Lan et al. used an inorganic sol–gel method to prepare high-dielectric-constant, isotropic spherical zirconia (yttria-stabilized) particles, as shown in Figure 8(a) [79]. They adhered a monolayer of microsphere onto tape and then poured PDMS into a stainless-steel container with the tape and microspheres at the bottom. After curing, the removal of PDMS yields a flexible metasurface composed of uniform yttria-stabilized zirconia microspheres with approximately 70 μm in diameter. The configuration allows for mechanical tunability of the electromagnetic response by stretching the PDMS. To enrich the tuning methods, they incorporated 80 μm diameter microceramic spheres (ZrO_2_) into a composite ink of nanostrontium titanate (STO) powder and PDMS [80]. STO, a ferroelectric material with thermally tunable properties, enables adjustment of the dielectric constant in the composite material.

In the aforementioned examples, microspheres are randomly distributed within PDMS. To achieve precise control over the positions of each unit cell, researchers have created 2D templates using 3D direct-writing methods. Gao et al. made ZrO_2_ microspheres with a diameter of 130 μm by reaction spray atomization method in Figure 8(b) [84]. They sprinkled them on a PDMS grid through 3D layer-by-layer printing. A 40 μm thick Kapton adhesive layer secured the embedded microspheres to the grid. The dielectric constant of Kapton (approximately 1.4) is significantly different from that of ZrO_2_ (dielectric constant of 32.5) and is relatively less sensitive to terahertz waves. Excess microspheres can be easily removed without contact with the adhesive layer. Here, the PDMS grid served as support for the microspheres. In other studies, researchers employed patterned stainless-steel templates to facilitate self-assembly.

Some researchers used thin stainless-steel templates with various geometric shapes to assist self-assembly, as shown in Figure 8(c). These templates are etched using solid-state lasers [81]. With blowing the microspheres from left to right with a soft slider, the microspheres are pushed into and secured within the template holes. After removing the templates and excess microspheres, controllable microsphere-based dielectric metasurfaces with the hexagonal arrangement are obtained. The different template changes the array structure and spacing of the microspheres, allowing for the exploration of a range of novel configurations. For example, Bi et al. fabricated various arrangements of microsphere metasurfaces, including dimers, trimers, tetramers, and chains, as shown in Figure 8(d) [82]. Self-assembly techniques offer a low-cost approach to manufacturing large-area terahertz dielectric metasurfaces, enabling the fabrication of ultra-large ZrO_2_ structures exceeding 900 × 900 cm^2^ (Figure 8(e)). Additionally, this method has been extended to other materials, such as Al_2_O_3_ microspheres.

### 3.3 Direct writing techniques

The direct writing techniques include various methods such as laser micromachining and 3D printing. They allow for real-time modifications of layouts, and precise control over solid micro- and nanostructures, enabling manipulation of electromagnetic waves in terahertz photonic crystals. Compared to traditional photolithography and etching processes, direct writing simplifies fabrication by eliminating the mask-making step, and it allows for easy alteration to mask design. Therefore, this technique is appropriate for the rapid development of solid structures and small-scale production. However, compared to photolithography with an exposure time of only tens of seconds, direct writing is more time-consuming and limited by the rheological properties of the materials, making it suitable for small-scale laboratory research. Here, we focus on direct-write micromachining and 3D printing techniques used for manufacturing terahertz gratings, which have demonstrated excellent Mie resonance performance and inspired new approaches in the design and fabrication of biosensors.

**Figure 9:**
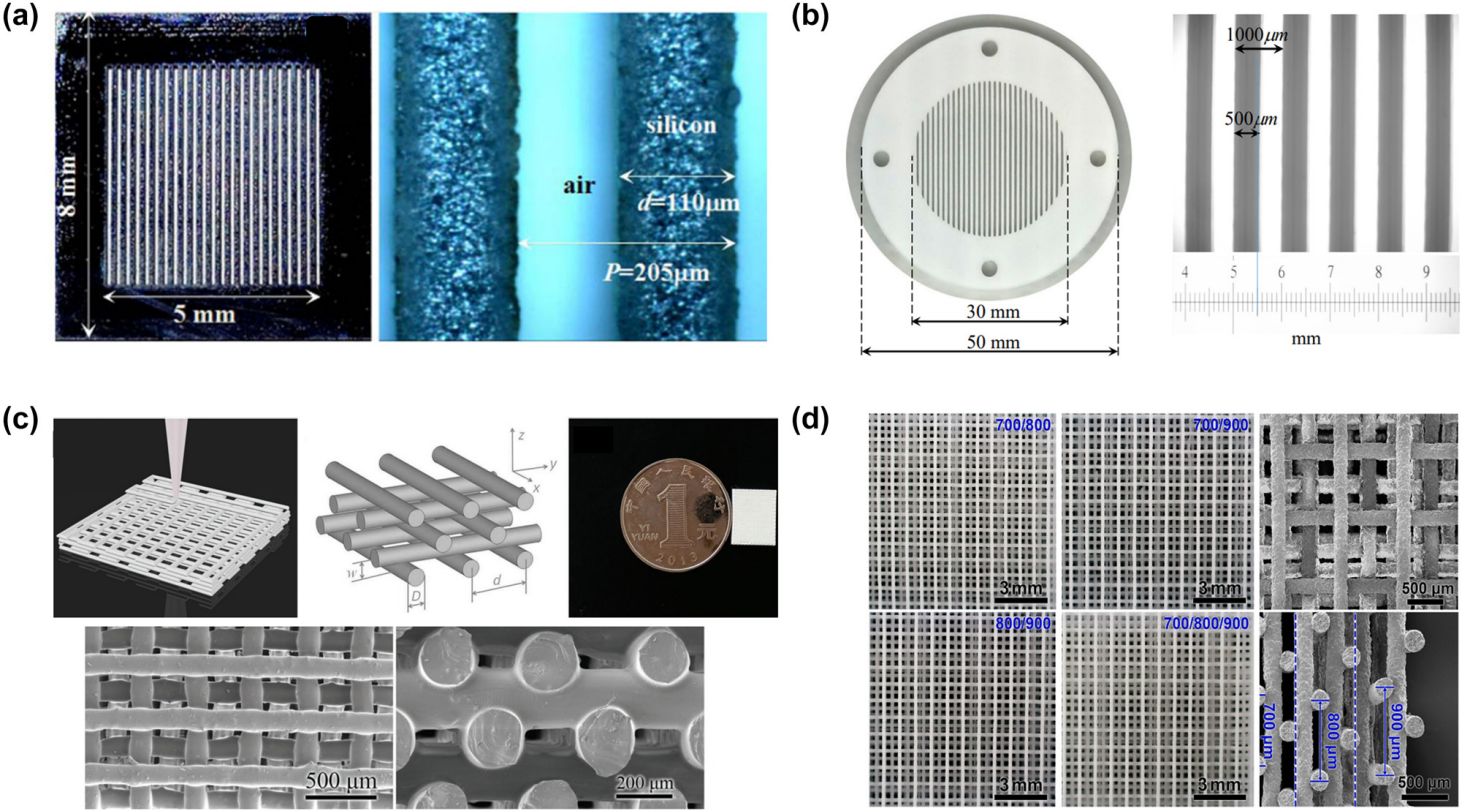
Fabrication techniques for dielectric gratings and photonic crystals. (a) Photograph of one-dimensional dielectric gratings fabricated by direct laser writing technology [85]. (b) Photograph of 1D planar photonic crystals by fused deposition modeling (FDM) of 3D printing [37]. (c) Flexible 3D terahertz photonic crystals with periodic cross-stacked structures printed layer by layer [86]. (d) The SEM image of terahertz photonic crystal heterostructures based on Al_2_O_3_/PDMS composite ink using direct ink writing 3D printing technology [87]. (a) Reproduced with permission. Copyright 2015, AIP Publishing Group. (b) Reproduced with permission. Copyright 2023, Optica Publishing Group. (c) Reproduced with permission. Copyright 2017, Wiley Publishing Group. (d) Reproduced with permission. Copyright 2023, Elsevier Publishing Group.

For instance, Yang et al. employed laser direct writing technology to fabricate one-dimensional dielectric gratings on double-side polished silicon wafers with a total size of 5 × 5 mm^2^, as illustrated in Figure 9(a) [85]. Using a solid-state nanosecond laser (Enpon-Nano-H532), they precisely cut parallel rectangular silicon rods with lengths, periods, widths, and thicknesses of 5,000, 205, 110, and 110 µm, respectively. Although the precision of laser direct writing is not as high as traditional photolithography and etching methods, it offers high design flexibility without the need for masks, enabling rapid and cost-effective patterning fabrication. Additionally, the one-dimensional dielectric gratings exhibit low absorption loss, making them ideal for developing frequency-selective and angle-independent perfect reflectors, as well as narrow bandwidth biosensors.

Beyond direct laser micromachining, additive manufacturing techniques such as 3D printing are also used to create one-dimensional gratings, the simplest 1D planar photonic crystals (PhCs) in Figure 9(b) [37]. Researchers fabricated 1D PhCs composed of dielectric rods by fused deposition modeling (FDM) of 3D printing. FDM is an additive manufacturing technique that involves heating and melting thermoplastic polymers, extruding them through a nozzle, and depositing targeted structures layer by layer. In this case, they used acrylonitrile butadiene styrene (ABS) with a high refractive index contrast as the printing polymer material. The high contrast gratings (HCGs) can excite sharp high-Q resonances, which can be used for high-sensitivity and low-cost terahertz sensors.

In addition to 1D gratings, more researchers employed various composite inks for the printing of 3D photonic crystals. These composite inks include TbFeO_3_ [88], mixtures such as barium titanate and PDMS, reduced graphene oxide/spherical carbonyl iron/PDMS [89], and Al_2_O_3_/PDMS [87]. PDMS is often chosen for its favorable properties, ensuring smooth flow through the nozzle under appropriate pressure. The rheological characteristics of PDMS prepolymers can be adjusted to meet specific nozzle requirements. For example, Zhu et al. designed a composite ink with barium titanate (BaTiO3) nanoparticles dispersed in PDMS [86]. They printed flexible 3D terahertz photonic crystals (3D-TPCs) with periodic cross-stacked structures layer by layer, as shown in Figure 9(c). The PDMS served as the flexible component, enabling mechanical tunability by altering the geometry of the 3D-TPCs. By varying the BaTiO_3_ nanoparticle content from 10 % to 40 %, the refractive index of the composite ink could be adjusted, thereby changing the resonant characteristics. In the printing process, the composite ink was loaded into a syringe and dispensed through a plastic nozzle. The deposition speed was kept at 5 mm/s, controlled by a pneumatic system. The pressure depends on the ink viscosity and extrusion rate. After that, the 3D-TPCs were cured at 80 °C for 2 h. They printed 3D-TPCs with 8, 12, and 16 layers, with rod spacings ranging from 300, 400, 500–600 µm. These structures exhibited significant photonic bandgap peaks in the terahertz frequency range, opening a new avenue for fabricating tunable sensors.

Additionally, Feng et al. reported the fabrication of terahertz photonic crystal heterostructures (TPCHs) based on Al_2_O_3_/PDMS composite ink using direct ink writing (DIW) 3D printing technology, as shown in Figure 9(d) [87]. By applying pressure to the syringe plunger with a pneumatic dispenser, the ink flow was controlled. They fabricated a series of TPCHs with different lattice constants, which exhibited unique photonic bandgaps such as ultra-wide bandgaps, dual bandgaps, or multiple bandgaps in the terahertz frequency range, showing great potential for multimode or multichannel optical devices. For 3D printing with direct writing technology, the dielectric materials of the composite ink system can be freely designed according to functional requirements, enhancing structural tunability. For instance, temperature-sensitive dielectric ceramic material TbFeO_3_ can be obtained via solid-state sintering and made into a composite ink with rheological properties for 3D printing [88]. These achievements enhance metasurface fabrication and broaden application prospects in areas such as stealth, sensors, and flexible wearable devices.

## 4 Multiscenario sensing methods and applications

Dielectric metasurfaces hold significant promise for terahertz sensing, providing high sensitivity, label-free detection, and real-time monitoring of biological and chemical processes. This section reviews recent advances in dielectric metasurfaces across diverse sensing applications, including biomolecule sensing, biomedical detection, environmental monitoring, and chiral sensing, highlighting their transformative potential in both scientific research and industrial applications.

### 4.1 Biomolecule detection assisted by chemical modification

**Figure 10:**
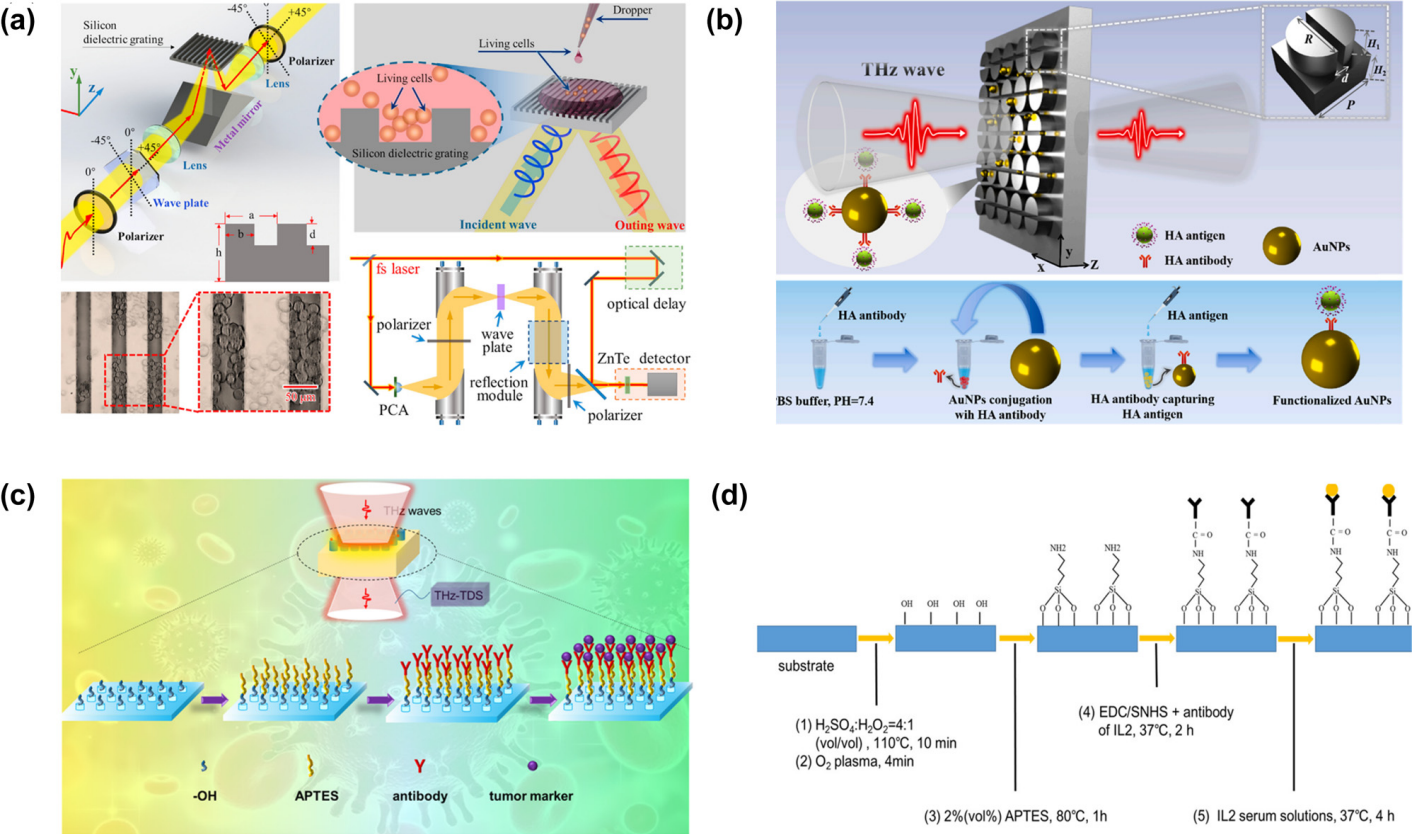
Dielectric metasurfaces for biomolecule detection. (a) Dielectric grating biosensors for quantitative detection of liver cancer cells [34]. (b) Dielectric metasurface functionalized with gold nanoparticles (Au NPs) to enhance the target biomolecule binding characteristics [35]. (c) Dielectric metasurface biosensor for the early diagnosis of ovarian and breast cancers [39]. (d) Dielectric metasurface biosensor for the early detection of cytokines [69]. (a) Reproduced with permission. Copyright 2021, Elsevier Publishing Group. (b) Reproduced with permission. Copyright 2022, Elsevier Publishing Group. (c) Reproduced with permission. Copyright 2023, ACS Publishing Group. (d) Reproduced with permission. Copyright 2024, Micromachines Publishing Group.

Dielectric metasurfaces for biomolecule detection can achieve enhanced sensitivity and selectivity when combined with chemical modifications. For example, Zhang et al. proposed a dielectric terahertz grating as a biosensor to enhance the response of analytes, as shown in Figure 10(a), avoiding strong water absorption through a reflection terahertz system [34]. By exciting extrinsic chirality with an incident angle of 30°, they detected the left- and right-handed polarized reflected wave with a pair of polarizers rotated by 90°. They realized quantitative detection of liver cancer cells and identification of cell types in a water environment and verified the anticancer activity of aspirin (Asp). In experiments, they cultured cancer cells with HepG2, Huh7, and H7402 liver cancer cells adherently and investigated apoptosis performance influenced by various concentrations of Asp from 1 mM to 4 mM. The terahertz CD spectra showed a linear decrease with the increasing Asp concentration. It is consistent with the trend of Asp to cell number, confirming Asp’s negative effect on liver cancer cells. The minimum detectable concentration was about 10^4^ cells/mL. This work has potential applications for evaluating cancer cell proliferation and developing candidate cancer therapeutics.

Although dielectric metasurface has significant advantages in biosensing due to its rich resonance modes, most previous studies focus on quantitative and nonspecific target detection. Achieving efficient specific sensing of low concentrations of molecules remains a challenge. Shi et al. (Figure 10(b)) [35] addressed this by integrating functionalized gold nanoparticles (Au NPs) with a dielectric metasurface to enhance the target biomolecule binding characteristics. The metasurface provides strong local field enhancement. The Au NPs were modified with anti-HA tag antibodies to achieve specific binding of the HA antigen, enabling specific immunosensing of the human influenza hemagglutinin tag protein (HA antigen) at nanomolar concentrations. The maximum detection accuracy reached 1.05 nmol/ml, with a sensitivity of 2.96 GHz ml/nmol–2.66 times higher than that of nonfunctionalized Au NPs. Furthermore, control experiments using bovine serum albumin (BSA), ovalbumin (OVA), and whey protein (WP) confirmed the specificity of the method, as these proteins showed no significant spectral shifts, indicating they did not bind to the HA antibody.

Another promising approach was demonstrated using a silicon pillar metasurface fabricated using glass and silicon wafer bonding and dry etching processes in Figure 10(c) [39]. The structure featured a large dielectric constant contrast between the silicon and the substrate, exhibiting a Q factor of 27 and two Mie resonances within the low-loss silicon pillars. The metasurface was functionalized with antibodies specific to tumor markers (CA125 and HER2) via oxygen plasma, APTES, and EDC/NHS/fetal bovine serum modifications, allowing for selective detection of ovarian cancer marker CA125 and breast cancer marker HER2, with detection limits of 1 ng/mL and 0.1 ng/mL, respectively. This label-free, real-time detection method demonstrated a linear response to marker concentrations ranging from 1 ng/mL to 10 μg/mL. Specificity tests confirmed that the antibodies successfully modified the device, and the respective tumor markers bound exclusively to their corresponding antibodies. The dielectric metasurface biosensor provides a promising sensing platform for the early diagnosis of ovarian and breast cancers.

Using a similar antibody functionalization process, Men et al. developed a dielectric metasurface sensor through bonding and dry etching for the specific detection of low concentrations of interleukin-2 (IL-2), as shown in Figure 10(d) [69]. The sensor achieved a detection limit of approximately 100 pg/mL for IL-2 with a Q factor of 35 at 0.82 THz. Surface modifications were characterized using X-ray photoelectron spectroscopy (XPS), confirming successful antibody modification through the formation of peptide and silane bonds. This approach enables the early detection of cytokines, offering the potential for diagnosing extreme immune responses.

Dielectric metasurfaces exhibit significant potential in biomolecule detection, particularly when functionalized with chemical modifications. The integration of functionalized nanoparticles and antibody-modified metasurfaces has demonstrated exceptional sensitivity and specificity in applications like cancer marker detection and cytokine monitoring. Although these technologies are not yet widely established as gold standards in clinical diagnostics, they represent promising tools for future disease diagnosis and monitoring. Further development of functionalization techniques and resonance optimization will be essential to transition these metasensors from research prototypes to practical clinical applications.

### 4.2 Biomolecule and biomedical sensing

**Figure 11:**
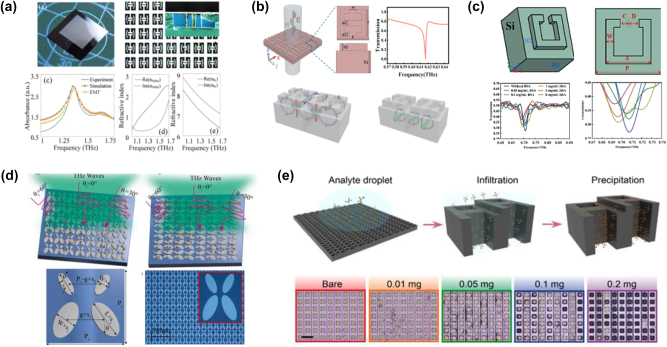
Dielectric metasurfaces for biomolecule detection. (a) All-silicon metasurface sensor for Bt protein detection [83]. (b) All-silicon metasurface biosensor for BSA detection [90]. (c) Another all-silicon metasurface biosensor for BSA detection [76]. (d) Dual-resonance biosensor for identifying biomolecules using THz fingerprint spectra [91]. (e) Dielectric metasurface THz biosensor for amino acids identification [38]. (a) Reproduced with permission. Copyright 2022, Optica Publishing Group. (b) Reproduced with permission. Copyright 2024, Elsevier Publishing Group. (c) Reproduced with permission. Copyright 2023, Elsevier Publishing Group. (d) Reproduced with permission. Copyright 2024, Elsevier Publishing Group. (e) Reproduced with permission. Copyright 2024, Elsevier Publishing Group.

Dielectric metasurfaces significantly enhance light–matter interactions, enabling ultra-sensitive detection of biomolecules. For instance, Cui et al. [83] developed an all-silicon metasurface sensor chip with two opposing T-shaped pillars, as a highly sensitive biosensor for *Bacillus thuringiensis* (Bt) protein, as shown in Figure 11(a). This sensor operates as a narrow-band absorber in the THz range, with a resonance layer and a substrate layer. In their experiments, they detected varying concentrations of Bt protein (from 5 to 500 ppm) by dripping solutions onto the sensor surface. The absorbance intensity increased and the resonance frequency redshifted as protein concentrations rose, showing strong linear correlations with high regression coefficients (0.8988 and 0.9238). This demonstrates the potential of the sensor for precise, trace-level protein detection.

In another study, Cui et al. [90] developed an all-silicon metasurface biosensor supported by magnetic toroidal dipole resonance under THz excitation, as shown in Figure 11(b). This metasurface exhibited a Fano resonance with a simulated high-Q factor of 272.84, due to the interaction between multiple magnetic ring dipoles in the structural layer and multiple magnetic dipoles in the substrate. The fabricated biosensor achieved a detection limit as low as 0.027 mg/mL and a sensitivity of 9.7 GHz/(ng/mm^2^) bovine serum albumin (BSA) detection. The actual Q factor of the bare biosensor reached 76.8, demonstrating significantly enhanced performance compared to other biosensors. These advancements highlight the ongoing trend toward greater precision and sensitivity in dielectric metasurface biosensors.

Yang et al. [76] proposed a high-Q factor square C-shaped all-silicon metasurface biosensor with obvious polarization sensitivity, as shown in Figure 11(c). They tested its performance using various concentrations of BSA (ranging from 0.05 mg/mL to 8 mg/mL) by applying the solution to the sensor and heating it to form a thin film. To quantify the characteristics of biosensors, they analyzed the *Q* factor (*Q*), sensitivity (*S*), and figure of merit (*FOM*) according to the formulas: *Q* = *f*∕*FWHM*, *S* = Δ*f*∕Δ*n*, *FOM* = *S*∕*FWHM*, where *f* is the resonance frequency, Δ*f* is the full-width at half-maximum (FWHM) of the resonance, and Δ*n* represents the concentration of the analyte. The results indicated that the resonance valley first increased, then decreased, and finally increased again as the BSA concentration rose, with a gradual redshift in resonance frequency. The detection limit for BSA was found to be 0.05 mg/mL, confirming the high sensitivity of the metasurface-based biosensor.

THz spectroscopy also provides a powerful tool for detecting biomolecules via molecular fingerprints. In Figure 11(d), Xu et al. [91] demonstrated a dual-resonance biosensor for identifying biomolecules and their concentrations based on unique THz fingerprint spectra under varying incident angles and polarization directions of THz waves. This method opens up new possibilities for biomedical molecule identification through spectral analysis. Similarly, Lin et al. [38] proposed a dielectric metasurface THz biosensor based on multiple controllable QBIC resonances, as shown in Figure 11(e). By manipulating the degree of longitudinal symmetry breaking, a high *FOM* of 15.2 was experimentally achieved, enabling the qualitative and quantitative identification of amino acids through strong light–matter interactions at hotspots. Further advancements include a planar THz metasurface sensing method proposed by Liu et al. [92], leveraging multiplexed quasi-BIC modes to achieve broadband molecular fingerprint detection with up to 330-fold enhancement for ultra-thin analytes through evanescent wave amplification. Another innovative approach by Liu et al. [93] introduced a single-pixel graphene metasurface that enables ultra-wideband THz fingerprint sensing (1.5 THz bandwidth) with a 17.4 dB enhancement and a limit of detection (LoD) of ≤0.64 μg/mm^2^, offering the potential for dynamic reconfigurable sensing and slow light modulation. Additionally, a pixelated frequencyagile metallic metasurface with graphene-integrated C-resonators [94] also demonstrated high sensitivity and broadband THz fingerprint detection. These recent advancements highlight the growing potential of dielectric metasurfaces for precise and sensitive detection of proteins, positioning them as powerful tools in the field of biosensing.

**Figure 12:**
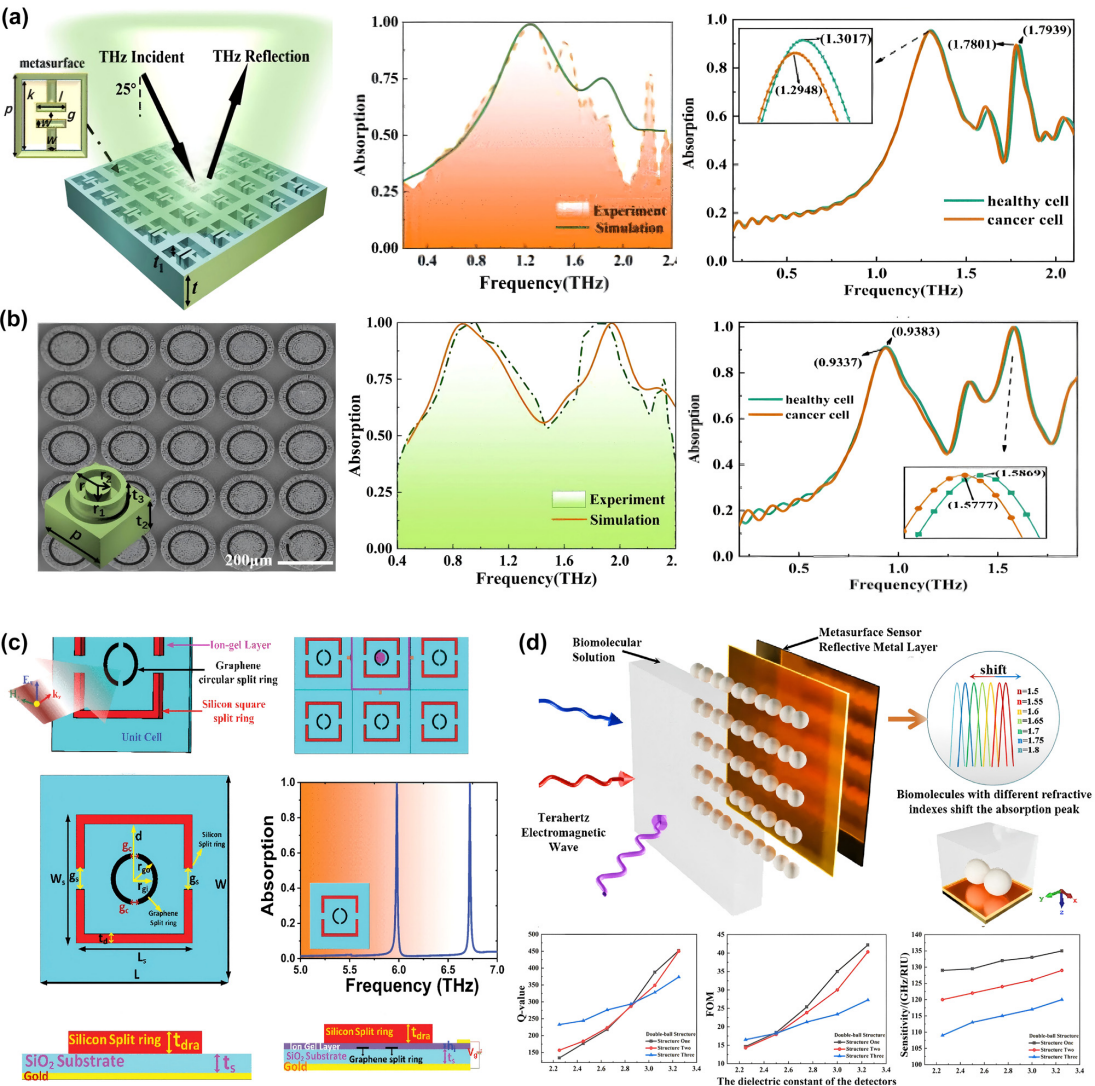
Dielectric metasurface for biomedical detection. (a) All-silicon dielectric absorber (AMTAs1) and its THz absorption spectrums of healthy and cancer cells [96]. (b) The other all-silicon dielectric absorber (AMTAs2) and its THz absorption spectrums of healthy and cancer cells [96]. (c) A dual-band biosensor based on SSSR and GCSR for malaria detection [97]. (d) A high-Q factor THz dielectric biosensor with zirconium oxide microspheres for amino acid detection [98]. (d) Reproduced with permission. Copyright 2024, Springer Nature Publishing Group.

Dielectric metasurface sensors also offer a promising platform for biomedical detection in the terahertz range [95]. They can successfully distinguish between cancerous and healthy cells by tracking the changes in resonance frequency or the transmission/reflection intensity. For example, Wang et al. [96] reported two dielectric THz absorbers patterned with different structures on all-silicon wafers, as effective refractive index sensors, as shown in Figure 12(a) and (b). The local field enhancement generated by the first absorber (called AMTAs1) is concentrated around parallel gaps, while in the second absorber (called AMTAs2), it is concentrated at both ends of the ring. Experimental results showed that both absorbers could differentiate between healthy and cancerous cells. The cancer cells caused a redshift in the response frequencies compared to healthy cells, with sensitivities of 0.4 THz/RIU and 0.81 THz/RIU for AMTAs1, and 0.59 THz/RIU for AMTAs2, respectively. These findings demonstrate the ability of dielectric metasurface for biomedical detection.

In another study, Patri et al. [97] designed a dual-band biosensor with perfect ultra-narrowband absorption at 5.98 THz and 6.72 THz, as shown in Figure 12(c). The biosensor consists of a silicon-based square split ring (SSSR) and a graphene-based circular split ring (GCSR). It can be used for malaria detection by monitoring the refractive index changes in red blood cells (RBCs) as the disease progresses. The sensor showed significant shifts in *S*, FOM, and *Q* values as the refractive index of RBCs varied from 1.383 to 1.373, with measurable changes in both the lower band (LB) and upper band (UB), such as *S* from 0.6666 to 0.6772, FOM from 42.1886 to 42.3275, and *Q* from 362.322 to 357.9625 in the LB, as well as *S* from 0.5308 to 0.5362, FOM from 35.3873 to 44.6828, and *Q* from 434.58 to 543.5 in the UB. These results demonstrated the sensor’s capability to accurately diagnose malaria based on changes in the electromagnetic response of infected RBCs.

To further improve the *Q* factor and the sensitivity of the dielectric metasurface biosensors, Gao et al. [98] proposed a high-Q factor THz dielectric biosensor with zirconium oxide microspheres. In Figure 12(d), these microspheres can boost strong Mie resonance, resulting in a high-*Q* factor of 451.87 and a theoretical sensitivity of 135 GHz/RIU, respectively. This design shows exceptional potential for detecting the refractive indices of biomolecules such as amino acids, presenting an innovative approach in metasurface-based biosensor development.

### 4.3 Environmental monitoring

Environmental monitoring has become a growing concern, leading to increased demand for tools capable of detecting pollutants, such as acetone and pesticide residues, with high sensitivity and speed. Traditional detection methods, including chemical analysis, instrumental analysis, and biomonitoring, each come with distinct advantages and limitations. Chemical analysis, for example, is cost-effective but time-consuming and operationally complex, whereas instrumental methods offer high sensitivity, high selectivity, and fast speed at the expense of costly equipment and procedures. In contrast, terahertz (THz) metasurfaces offer a competitive platform for environmental monitoring due to their high sensitivity, low analyte dosage requirements, fast response times, and portability. Their simple predetection procedures and cost-effectiveness make them promising candidates for pollution detection in a range of scenarios.

Pesticides, while widely used in agriculture for pest control, sterilization, and weeding [99], pose significant risks when excessively used, as they leave residues that contaminate soil and water, ultimately harming both the environment and human health [100]. Common methods for detecting pesticide residues, such as gas chromatography and high-performance liquid chromatography, are expensive and time-consuming. In this context, terahertz metasurface sensors present a compelling alternative.

**Figure 13:**
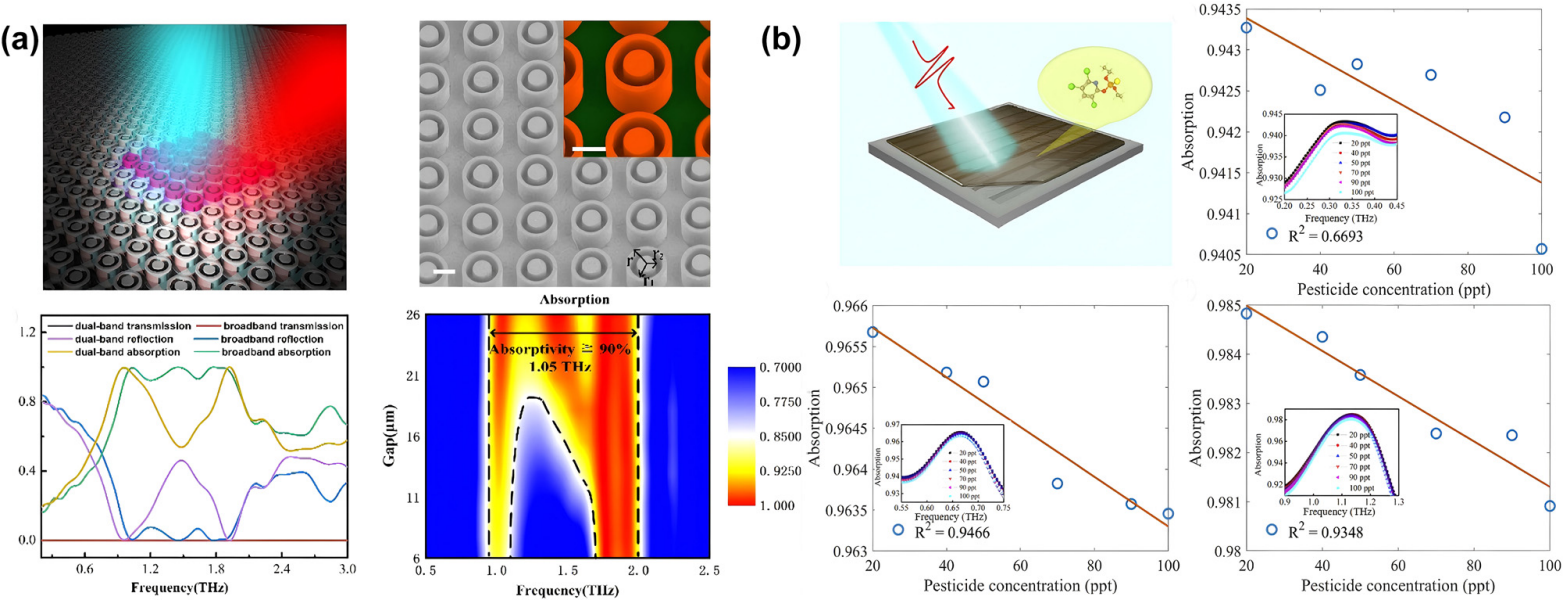
Dielectric metamaterial absorbers for the chlorpyrifos detection. (a) Dielectric THz metamaterial absorbers and their broadband and dual-band absorption spectra [101]. (b) Tri-band metasurface absorber and its measured absorption spectra for 20–100 ppt of chlorpyrifos [33]. (a) Reproduced with permission. Copyright 2019, ACS Publishing Group. (b) Reproduced with permission. Copyright 2021, Optica Publishing Group.

In 2019, Yue et al. [101] demonstrated two types of dielectric metamaterial absorbers made of patterned dielectric resonators for pesticide detection. As illustrated in Figure 13(a), the absorbers consist of a square array of dielectric rings and cylindrical disks on highly N-doped silicon. The two designs demonstrate different absorption properties due to their different geometries although with the same carrier density. The first design is a broadband absorber, achieving more than 90 % absorption between 0.95 and 2.0 THz under the 25° angle of incidence. In contrast, the second design operates as a dual-band absorber, demonstrating two discrete absorption peaks at 0.96 THz and 1.92 THz, with both absorption peaks exceeding 99.6 %. It was demonstrated that when chlorpyrifos (CPS) was deposited on the metasurface absorbers, a clear shift in the absorption spectra occurred as CPS concentrations varied from 0.1 to 100 ppm. In particular, the dual-band absorber demonstrated greater sensitivity due to its higher Q factors. These findings confirm the feasibility of using THz dielectric metasurface for high-sensitivity pesticide detection.

In 2021, Yue et al. [33] presented a perfect metasurface absorber (PMA) sensor based on silicon, which achieves quad-band or tri-band perfect absorption, as shown in Figure 13(b). The average absorption amplitude reaches 96.1 % at the resonance frequencies and the maximum quality factor reaches 12.64 and 12.64, respectively. In experiments, this absorber sensor exhibits a high selectivity of pesticides in which chlorpyrifos is prepared at different concentrations (20, 40, 50, 70, 90, and 100 ppt). The absorption spectra show that the resonance amplitudes gradually decrease as the chlorpyrifos concentration rises. In comparison, the last two absorption peaks demonstrate a linear relationship with concentration, showing them as reliable indicators for detecting the concentration of chlorpyrifos. These advancements demonstrate the potential of dielectric metasurfaces for efficient, cost-effective monitoring of organic pollutants and pesticide residues, paving the way for more accessible environmental sensing technologies.

### 4.4 Chiral sensing

Chirality, a fundamental property of matter, refers to the inability to superimpose an object with its mirror image through translation and rotation [102], [103]. Enantiomers, which are chiral molecules, are prevalent in various biological structures and serve as the building blocks of life. Many essential biological molecules, including amino acids, sugars, proteins, and drugs, exhibit chirality, with distinct left-handed (sinister, s-) and right-handed (dexter, d-) enantiomers [104]. While enantiomers share identical molecular weights and chemical compositions, their biological activities can differ drastically [105]. For instance, the sinister form of thalidomide is an effective sedative, whereas its dexter form is teratogenic, highlighting the critical importance of distinguishing enantiomers in biology, pharmacology, and toxicology [106].

In a chiral medium, electromagnetic coupling phenomena result from interactions with circularly polarized light (CPL), which allows for the detection of enantiomers through circular dichroism (CD) spectroscopy. CD measures the differential absorption of left- and right-circularly polarized light and is expressed as *CD* = *A*_*LCP*_ – *A*_*RCP*_, where *A*_*LCP*_ and *A*_*RCP*_ represent the absorption intensities of left- and right-circularly polarized light, respectively. However, many natural chiral biomolecules exhibit weak optical chiral responses, especially in the terahertz range, where CD signal intensities are significantly reduced compared to the ultraviolet (UV) and visible ranges [107].

Metasurfaces offer a promising solution to this limitation by generating superchiral fields, which enhance the interaction between light and chiral molecules. The optical chirality (*C*) of the superchiral fields is defined by C=−ω2c2ImE*⋅H=−ω2c2EHcosφiE,H [108]. These superchiral fields, with large values of *C*, are capable of significantly amplifying the optical response of chiral molecules.

In recent years, metasurfaces have been widely used in optical chiral sensing applications. Through the design of specific nanostructures that can form localized hotspots of suprachiral light fields, the interaction between light fields and chiral molecules can be significantly enhanced, leading to an increase in the sensitivity of optical chiral sensing. While plasmonic metasurfaces can generate highly enhanced electric fields, their magnetic resonances are often weak or spectrally separated from the electric resonances, leading to spatially nonuniform superchiral fields and restricting the overall chiral response [108]. In contrast, dielectric metasurfaces not only support both strong electric and magnetic resonances but also exhibit reduced dissipation and lower thermal conductivity. With tailored structural designs, dielectric metasurfaces can produce single-sign, uniform superchiral hotspots with significantly enhanced volume-averaged optical chirality, making them more effective for chiral sensing applications [109]. These advantages open new avenues for developing high-sensitivity terahertz chiral metasensors.

**Figure 14:**
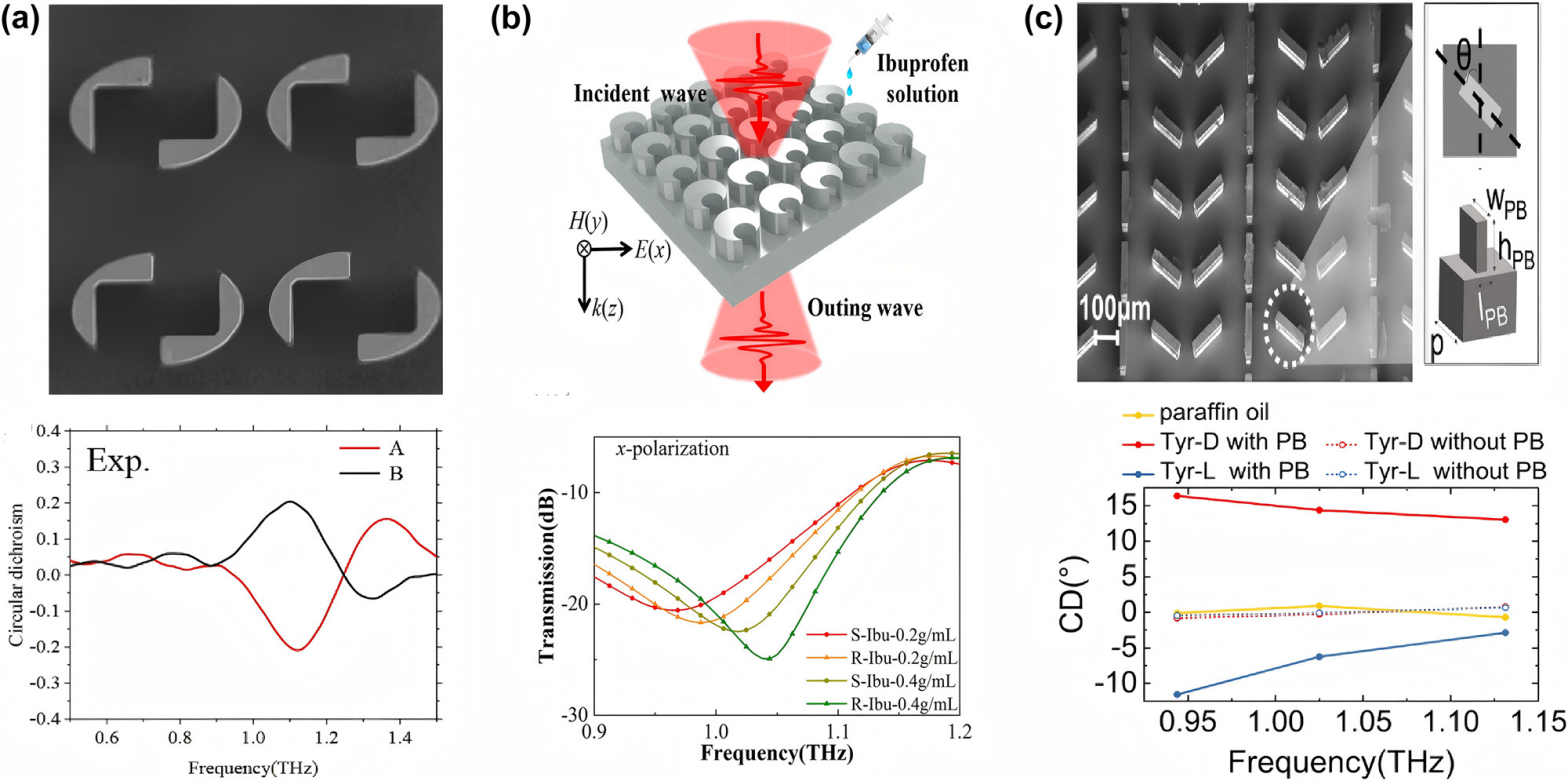
Structural chiral dielectric metasurface. (a) L-type dielectric metasurface [110]. (b) Crescent cylinder metasurface for chiral ibuprofen isomers distinguishing [111]. (c) PB metasurface for D- and L-tyrosine identification [112]. (a) Reproduced with permission. Copyright 2021, Optica Publishing Group. (b) Reproduced with permission. Copyright 2021, MDPI Publishing Group. (c) Reproduced with permission. Copyright 2023, Optica Publishing Group.

Dielectric metasurface can be categorized into two main types: structural chiral and achiral. In 2021, Li et al. proposed an L-type dielectric chiral metasurface, achieving terahertz CD values of 69.4 % theoretically and 43 % experimentally, as shown in Figure 14(a) [110]. In the same year, Shi et al. demonstrated a terahertz crescent circular array metasurface for the recognition of chiral ibuprofen isomers, as shown in Figure 14(b), showing excellent sensitivity of 60.42 GHz/mg in the range of 0.6–1.46 THz [111]. In another recent example, in 2023, Liu et al. presented a Pancharatnam–Berry (PB) metasurface based on high-resistance Si, as shown in Figure 14(c), which achieved 9.3-fold and 11.9-fold enhancement in CD values for *D*- and *L*-tyrosine to 16.4° and −11.6°, respectively [112]. These structural chiral dielectric metasurfaces can substantially enhance the chiral response and are promising for detecting and distinguishing enantiomers. However, such chiral metasurfaces often inhibit inherent background chirality signals, which may interfere with the sensitivity and accuracy of chiral sensing applications.

**Figure 15:**
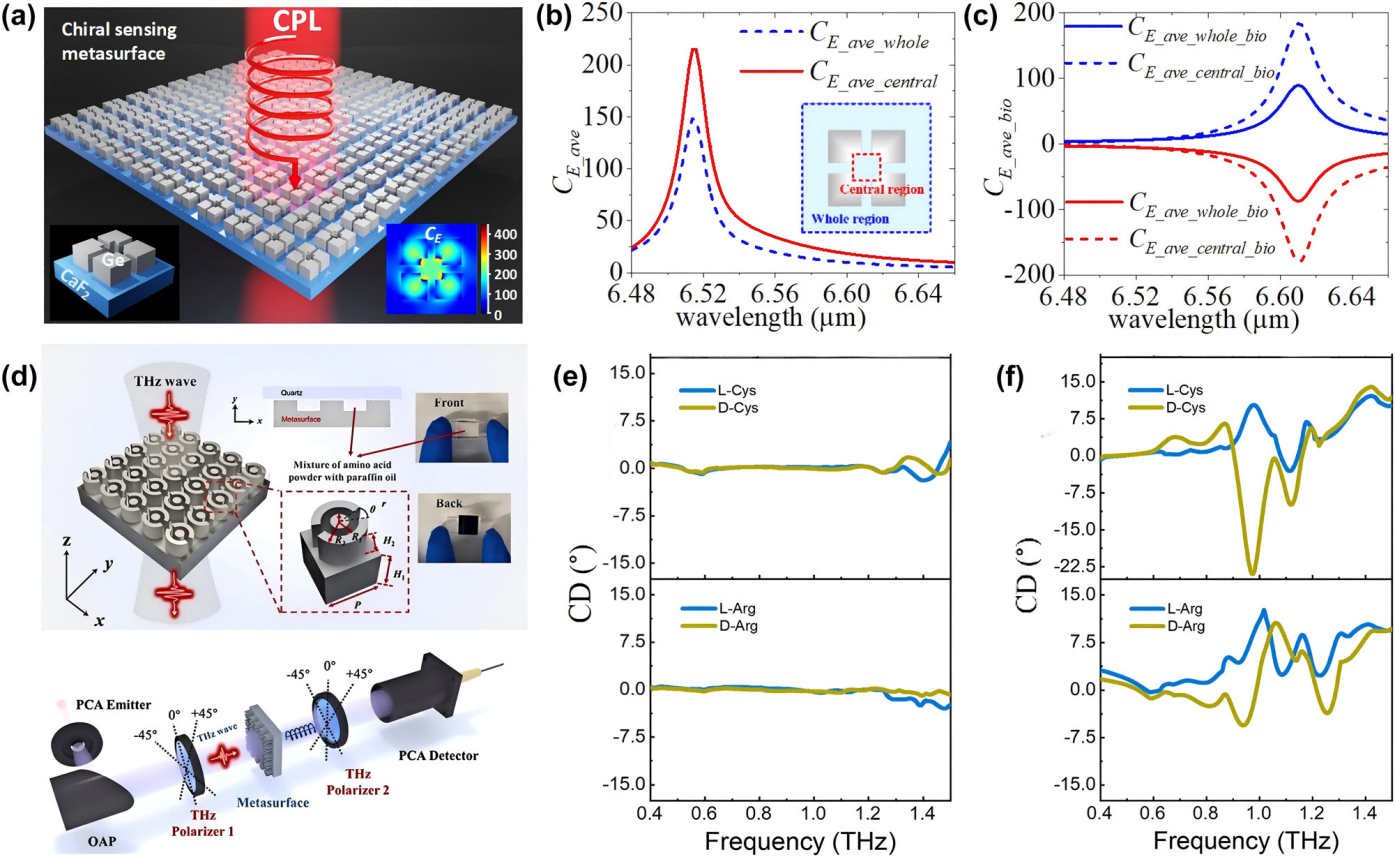
Structural achiral dielectric metasurfaces for chiral sensing. (a) Schematic of dielectric metasurface based on Ge tetramer nanoresonators [115]. (b) Chirality enhancement of the bare metasurface in the mid-infrared range [115]. (c) Chirality enhancement of the metasurface with biolayer in the mid-infrared range [115]. (d) Schematic of the dielectric metasurface for recognition of amino acid chiral enantiomers (top) and the THz-TDPS setup (bottom). (e) Experimental CD spectra for L- and D-Tyr without metasurfaces in the THz range. (f) Experimental CD spectra for L- and D-Tyr with metasurfaces in the THz range [118]. (a)–(c) Reproduced with permission. Copyright 2023, ACS Publishing Group. (d)–(f) Reproduced with permission. Copyright 2022, Elsevier Publishing Group.

To overcome the issue of background chirality signals, achiral dielectric metasurfaces with high-refractive-index materials have emerged as a promising platform for enhanced chiral sensing. Achiral dielectric metasurfaces, such as silicon nanodisk arrays [113], [114], holey silicon disks [108], Ge dual-dimer and tetramer nanoresonators [115], [116] (Figure 15(a)–(c)), and biperiodic diamond disks [117], have demonstrated for significant optical chirality enhancement in the mid-infrared, visible, and ultraviolet ranges. In the terahertz range, Chang et al. [118] utilized terahertz time-domain polarization spectroscopy in conjunction with a dielectric metasurface to amplify the chiral spectra of amino acids, as shown in Figure 15(d)–(f). The study demonstrated the dielectric metasurface enhanced CD signals for three pairs of amino acid enantiomers, with L-arginine achieving a maximum chirality enhancement factor of 97.15 at around 1 THz. Additionally, L- and D-cysteine exhibited a maximum CD difference of 34.22°, representing an 11.4-fold increase in chirality signal compared to nonmetasurface enhanced samples [118]. These advances demonstrate that dielectric metasurfaces hold great potential for high-performance, cost-effective chiral sensing, opening new avenues in terahertz biophysics and biochemistry.

## 5 Conclusion and outlook

Dielectric metasurfaces are emerging as a powerful alternative for a wide range of sensing applications. Offering lower electromagnetic losses, reduced heat generation, and higher Q factors compared to traditional metallic metasurfaces, they demonstrate remarkable potential for high-sensitivity detection. Constructed from biocompatible materials like silicon, dielectric metasurfaces provide excellent chemical stability in biological environments, enabling highly sensitive, label-free, and noninvasive detection. This allows for real-time monitoring of dynamic biological processes, including molecular reactions, cellular metabolism, and drug interactions, without the need for chemical or fluorescent labels. As a transformative platform, dielectric metasurface-assisted terahertz sensing holds immense potential in various fields and is poised to play a pivotal role in the next generation of terahertz sensing technology.

**Figure 16:**
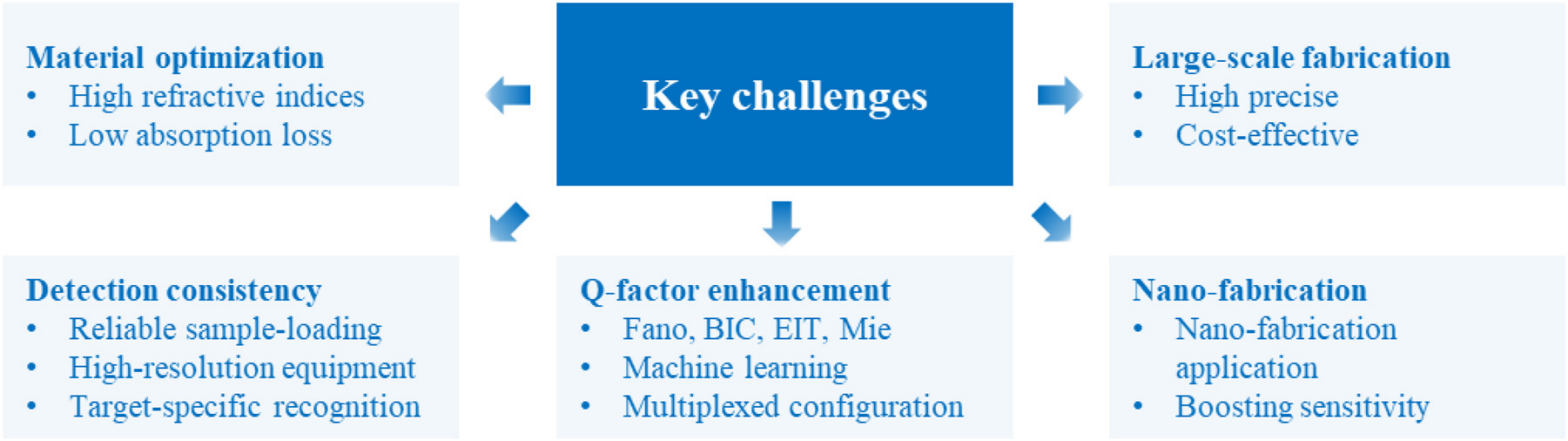
Key challenges faced by dielectric metasensors and potential solutions.

However, several challenges remain before this technology can achieve widespread practical implementation, as summarized in Figure 16: (1) Material Optimization. One key challenge remains in identifying dielectric materials that combine high refractive indices with minimal absorption loss. Achieving this balance is crucial to further improve the Q factor and enhance detection sensitivity. (2) Large-Scale Fabrication. Most current dielectric metasurfaces have demonstrated success at the lab but are not yet ready for mass production. Future efforts should focus on precise, cost-effective fabrication techniques, including innovative bonding methods and alternative low-refractive-index or flexible substrates, to support high-precision manufacturing and widespread adoption. (3) Detection Consistency. Despite the promise of dielectric metasurface sensors in detecting trace amounts of pesticides, cancer cells, and biomolecules, achieving consistent and reproducible detection remains a challenge. Contributing factors include environmental variability, fabrication imperfections, uneven sample distribution, and equipment limitations. Developing reliable sample-loading methods like microfluidics or spray deposition, and high-resolution measurement tools, alongside incorporating selective target-specific recognition mechanisms, will be essential for enhancing sensor accuracy and consistency in complex environments. (4) Q Factor Enhancement. Improving the Q factor remains a key focus for optimizing the performance of terahertz dielectric metasurfaces. Innovations such as incorporating new mechanisms like Fano, BIC, EIT, and Mie resonances, leveraging advanced design methodologies using machine learning, and employing multiplexed metasurface configurations are promising directions for future research and development. (5) Boosting Sensitivity with Nanofabrication: Nanofabrication techniques with nanometer-scale resolution can significantly enhance the sensitivity of THz metasurfaces by incorporating nanogaps that amplify light–matter interactions. For instance, virus-sized gold nanogaps with a width of 20 nm have been demonstrated for ultra-sensitive virus detection [119], [120]. These strategies provide valuable insights for developing dielectric metasensors with extreme light–matter interaction capabilities near nanogaps, opening new avenues for further sensitivity enhancements.

As dielectric metasurface-assisted terahertz sensing advances, it holds great promise for revolutionizing real-time, label-free, and noninvasive sensing solutions. The integration of THz metasurfaces with capabilities for wave generation [121], manipulation [11], and molecular sensing could pave the way for ultra-compact, portable devices ideal for rapid diagnostics and bioanalytical research. With ongoing innovation in materials, design, fabrication, and detection techniques, this emerging technology has the potential to drive breakthroughs across healthcare, medicine, environmental monitoring, and agriculture. Continued research and development will move it closer to practical, widespread applications, potentially transforming precision sensing across multiple scientific and industrial fields.
